# An optimised YOLOv4 deep learning model for efficient malarial cell detection in thin blood smear images

**DOI:** 10.1186/s13071-024-06215-7

**Published:** 2024-04-16

**Authors:** Dhevisha Sukumarran, Khairunnisa Hasikin, Anis Salwa Mohd Khairuddin, Romano Ngui, Wan Yusoff Wan Sulaiman, Indra Vythilingam, Paul Cliff Simon Divis

**Affiliations:** 1 Department of Biomedical Engineering, Faculty of Engineering, Universiti Malaya, Kuala Lumpur, Malaysia; 2 Department of Electrical Engineering, Faculty of Engineering, Universiti Malaya, Kuala Lumpur, Malaysia; 3 Department of Para-Clinical Sciences, Faculty of Medicine and Health Sciences, Universiti Malaysia Sarawak, Sarawak, Malaysia; 4 Department of Parasitology, Faculty of Medicine, Universiti Malaya, Kuala Lumpur, Malaysia; 5 Malaria Research Centre, Faculty of Medicine and Health Sciences, Universiti Malaysia Sarawak, Kota Samarahan, Sarawak Malaysia; 6 Center of Intelligent Systems for Emerging Technology (CISET), Faculty of Engineering, Universiti Malaya, 50603 Kuala Lumpur, Malaysia

**Keywords:** Malaria, YOLOv4, Optimised, Residual network, Residual block, Object detection

## Abstract

**Background:**

Malaria is a serious public health concern worldwide. Early and accurate diagnosis is essential for controlling the disease’s spread and avoiding severe health complications. Manual examination of blood smear samples by skilled technicians is a time-consuming aspect of the conventional malaria diagnosis toolbox. Malaria persists in many parts of the world, emphasising the urgent need for sophisticated and automated diagnostic instruments to expedite the identification of infected cells, thereby facilitating timely treatment and reducing the risk of disease transmission. This study aims to introduce a more lightweight and quicker model—but with improved accuracy—for diagnosing malaria using a YOLOv4 (You Only Look Once v. 4) deep learning object detector.

**Methods:**

The YOLOv4 model is modified using direct layer pruning and backbone replacement. The primary objective of layer pruning is the removal and individual analysis of residual blocks within the C3, C4 and C5 (C3–C5) Res-block bodies of the backbone architecture’s C3-C5 Res-block bodies. The CSP-DarkNet53 backbone is simultaneously replaced for enhanced feature extraction with a shallower ResNet50 network. The performance metrics of the models are compared and analysed.

**Results:**

The modified models outperform the original YOLOv4 model. The YOLOv4-RC3_4 model with residual blocks pruned from the C3 and C4 Res-block body achieves the highest mean accuracy precision (mAP) of 90.70%. This mAP is > 9% higher than that of the original model, saving approximately 22% of the billion floating point operations (B-FLOPS) and 23 MB in size. The findings indicate that the YOLOv4-RC3_4 model also performs better, with an increase of 9.27% in detecting the infected cells upon pruning the redundant layers from the C3 Res-block bodies of the CSP-DarkeNet53 backbone.

**Conclusions:**

The results of this study highlight the use of the YOLOv4 model for detecting infected red blood cells. Pruning the residual blocks from the Res-block bodies helps to determine which Res-block bodies contribute the most and least, respectively, to the model’s performance. Our method has the potential to revolutionise malaria diagnosis and pave the way for novel deep learning-based bioinformatics solutions. Developing an effective and automated process for diagnosing malaria will considerably contribute to global efforts to combat this debilitating disease. We have shown that removing undesirable residual blocks can reduce the size of the model and its computational complexity without compromising its precision.

**Graphical Abstract:**

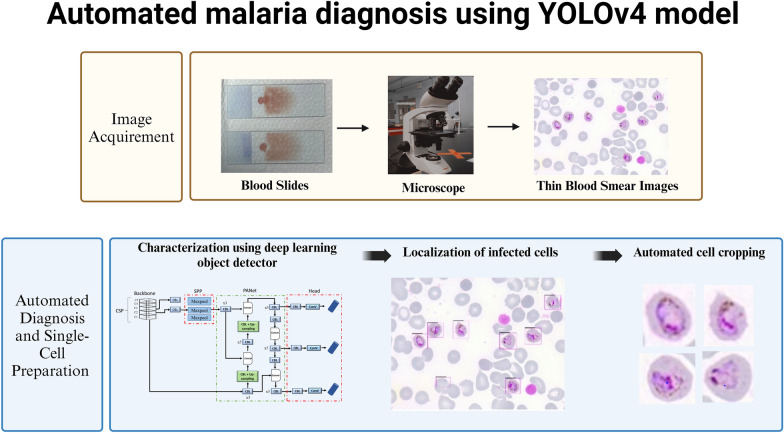

## Background

Malaria eradication has been a long-standing stated goal of the WHO since 1955. However, despite all efforts, malaria remains a significant global public health issue. A total of 247 million cases of malaria and 619,000 deaths attributable to malaria were reported globally in 2021 compared to 625,000 deaths in the first year of the COVID-19 pandemic (2020) [1, 2]. Between 2019 and 2021, the number of malaria cases continued to rise, albeit at a slower rate, from 245 to 247 million cases between 2020 to 2021 compared to the 2019–2020 period. In 2021, sub-Saharan Africa accounted for 95% and 96% of the cases and deaths attributable to malaria, respectively, with 80% of the deaths among children aged < 5 years. The existing trend in malaria cases challenges the WHO’s Global Technical Strategy for malaria 2016–2030, which aims to decrease the occurrence of malaria cases and the fatality rate by a minimum of 75% and 95% by 2025 and 2030, respectively, compared to the statistics recorded in 2015.

In addition to the* Plasmodium* species that are considered to cause malaria exclusively in humans, there has been an emergence of zoonotic malaria cases caused by the *Plasmodium knowlesi* over the years. The increase in malaria cases caused by *P. knowlesi* has caused notable concern, especially in the southeast Asian countries of Malaysia, Thailand and Indonesia, and there is a high risk of this species spreading globally through travel and tourism. Although this zoonotic malaria parasite was first discovered in monkeys, it can cause severe and swift outbreaks of sickness in humans, with a 1–2% fatality rate. A total of 2768 *P. knowlesi* cases were recorded worldwide in 2022, with Malaysia continuing to be the leading country as contributor of *P. knowlesi* cases, followed by Thailand and Indonesia; these three countries account for 90.5%, 3.1% and 0.1% of cases worldwide, respectively. Notably, the spread of this malaria parasite by monkeys has become a major public health concern, namely in the Borneo region of Malaysia [1, 5]. The increasing trend in the number of *P. knowlesi* infections in Thailand and Indonesia infections compared to 2021 is pronounced, despite Malaysia reporting its greatest number of *P. knowlesi* infections in 2022.

The Borneo region of Malaysia, which is known for its extensive forest cover, has documented the highest incidence of zoonotic malaria infections globally. The deforestation of these forests has unintentionally heightened the likelihood of encounters between zoonotic malaria hosts and humans, thereby increasing the transmission of *P. knowlesi* [6, 7]. Between 2008 and 2018, the number of *P. knowlesi* infections increased from 376 to 4131 cases, highlighting the urgency of the situation [7]. Despite a minor decrease to 3213 and 2609 cases in 2019 and 2020, respectively [1], there was a subsequent increase to 3575 cases in 2021 and 2500 cases in 2022.

There are specific obstacles to eradicating malaria because of the rise in *P. knowlesi* cases and transmission. Currently, nations that have eradicated the transmission of all four* Plasmodium* species known to cause malaria in humans are certified as being malaria-free. Nevertheless, the increasing number of *P. knowlesi* cases complicates attempts to eradicate malaria and has an impact on the WHO’s certification process for malaria-free status, which traditionally took into account only these four species recognised to be human-only malaria parasites. For example, Malaysia is still not certified as a malaria-free country due to the emergence of *P. knowlesi* cases, although it has reported zero infections by these four human-only* Plasmodium* species for 4 consecutive years. In light of the emergence of *P. knowlesi*, the WHO is reviewing its certification requirements.

In this context, automated detection and monitoring of possible outbreaks is critical given the growing threat posed by zoonotic malaria infections. In such countries as Malaysia, Thailand and Indonesia, the spread of zoonotic malaria, especially cases due to *P. knowlesi* has raised serious public health concerns. These cases have the potential to rapidly spread throughout the world, posing a major risk to public health. The increase in *P. knowlesi* infections highlights the significance of taking preventative action to identify and contain these outbreaks, particularly in Malaysia's Borneo region with its substantial forest cover. Given the challenges in eradicating malaria due to the surge in *P. knowlesi* cases, it is imperative to implement real-time identification strategies to effectively manage and control zoonotic malaria transmission.

### Current malaria diagnosis practices

Microscopic examination is the gold standard in malaria diagnosis [3] due to its cost effectiveness. However, while blood smear microscopy is the gold standard of malaria diagnosis set by WHO, it requires specialised training, which restricts its application in regions where malaria is prevalent. The main challenge in performing microscopic examinations is that the results are highly dependent on the skill of the microscopist. In malaria-endemic countries, the lack of resources is a significant barrier to a reliable and timely diagnosis. Often, healthcare personnel are more likely to be undertrained and underequipped and also divide their attention for malaria among other severe infectious diseases [4]. The need for more expertise in rural areas hinders diagnosis and treatment, leading to longer diagnosis times. The situation may not be better in a non-endemic country, however, where the disease is rarely seen, as the expertise level may not be maintained over the years, causing unfamiliarness with the disease. Therefore, continuous maintenance and enhancement of the skills of microscopists is crucial for accurate malaria diagnosis. Alternative diagnostic techniques could supplement the current diagnosis techniques and surmount their limitations.

In addition, in response to the challenges faced by traditional diagnostic methods, there has been a growing interest in leveraging advanced object detection models for malaria diagnosis. These models, constructed using deep learning algorithms, have demonstrated remarkable capabilities for detecting and classifying malaria-infected red blood cells (RBCs) with high accuracy and efficiency. When object detection models are used to analyse digital images of thin blood smears, they automatically identify and localise infected cells, thereby streamlining the diagnostic process and reducing the reliance on skilled personnel. Furthermore, the integration of object detection models into diagnostic workflows has the potential to enhance diagnostic accuracy, particularly in regions where access to trained microscopists is limited. Moreover, these models can operate autonomously, enabling continuous monitoring and rapid detection of malaria cases, even in remote or resource-constrained settings. As such, the adoption of object detection models represents a promising approach to augmenting current diagnostic practices and advancing the fight against malaria on a global scale.

### Application of deep learning convolutional neural networks in malaria diagnosis

In the realm of diagnosing malaria, there has been an increasing trend since 2016 to use deep learning approaches [14–18]. A review of the literature revealed that while many studies have focused on applying deep learning to malaria diagnosis, this focus revolves around using single-cell images to perform the classification of RBCs as infected or non-infected. For this classification problem, convolutional neural networks (CNN) mostly utilised to categorise malaria cells as either infected or non-infected [18–29]. In most studies, various deep learning approaches were employed to identify the best CNN model and method for classifying the cells. Therefore, in each study, a variety of CNN models were trained, and then hyperparameter tuned. A summary of the related studies that employed CNN are tabulated in Table 1.

**Table 1:** Summary of recent deep learning approaches in automated malaria diagnostic systems

Author	Database	*Plasmodium* species	Classification	Technique	Results
Sriporn et al. [18]	NLM, 7000 cell images	*P. falciparum*	Binary	Xception, Inception-V3,ResNet-50,NasNetMobile,VGG-16, AlexNet	Best performing model: Xception Accuracy: 99.28% Precision: 99.29% Recall: 99.29% F1-Score: 99.28%
Umer et al. [19]	NLM, 27558 cell images (150 infected, 50 healthy patients)	*P. falciparum*	Binary	Customised CNN	Accuracy: 99.96% Precision: 100% Recall: 99.93%
Zhao et al. [20]	NLM, 27558 cell images (150 infected, 50 healthy patients) Broad Institute dataset contains 1364 blood smear images with 80,000 infected cells	*P. falciparum* *P.vivax*	Binary	ResNet50V2, VGG16, VGG19, InceptionV3, DenseNet121, MobileNetV2	Best performing model: VGG-16 Accuracy: 96.53% Sensitivity: 95.0% Specificity: 98.07% AUC: 99.40% F1-Score: 96.48% MCC: 93.30% **Cross-dataset:** AUC:94.5%
Ragb et al. [21]	NLM, 27,558 cell images (150 infected, 50 healthy patients)	*P. falciparum*	Binary	SqueezeNet, MobileNetV2, GoogleNet, ResNet18, DarkNet19, InceptionV3, AlexNet, Xception, AlexNet, DenseNet201, ResNet101, VGG19, Ensembled model	Best performing model: Ensembled model Sensitivity: 97.94% Specificity: 97.78% Precision: 97.8%
Cinar et al. [22]	NLM, 27558 cell images (150 infected, 50 healthy patients)	*P. falciparum*	Binary	AlexNet, ResNet50, DenseNet201, VGG19, GoogleNet, InceptionV3	Best performing model: DenseNet201 Accuracy: 97.83%
Maqsood et al. [23]	NLM, 27558 cell images (150 infected, 50 healthy patients)	*P. falciparum*	Binary	VGG16, VGG19, Xception, Densenet121, Densenet169, Densenet201, Inceptionv3, InceptionResnet_v2, Resnet50, Resnet101, Resnet152, SqueezeNet, Customised CNN	Specificity: 97.78% sensitivity: 96.33% Precision: 96.82% Accuracy: 96.82% F1-Score: 96.82% MCC: 93.64%
Diyasa et al. [24]	NLM, 27,558 cell images (150 infected, 50 healthy patients)	*P. falciparum*	Binary	GoogleNet	Accuracy:93.89%
Loddo et al. [25]	NLM, 27558 cell images (150 infected, 50 healthy patients) MP-IDB, 229 images	*P. falciparum* *P. vivax*	Binary and Multi-class	AlexNet, DenseNet-201, ResNet-18, ResNet-50, ResNet101, GoogleNet, ShuffleNet, SqueezeNet,MobileNetV2, Inceptionv3, VGG-16	Best performing models Binary: ResNet-18 Accuray: 97.68% Multi-class: DenseNet-201: Accuracy: 99.40% **Cross-dataset validation:** Accuracy: 97.45%
Shambhu et al. [26]	NLM, 27558 cell images (150 infected, 50 healthy patients)	*P. falciparum*	Binary	Customised CNN	96.02%
Vijayalaskhmi et al. [27]	1030 infected images and 1520 non-infected images	*P. falciparum*	Binary	LeNet-5, AlexNet, GoogleLeNet, VGG16, VGG19	Best performing model: VGG19 Sensitivity: 93.44% Specificity: 92.92% Precision: 92.92% Accuracy: 93.13% F1-Score: 93.13%
Arshad et al. [28]	IML-malaria	*P. vivax*	Multi-class	VGG16, VGG19, ResNet50V2, DenseNet169, DenseNet201	Best performing model: ResNet-50v2: 79.61%
Rahman et al. [29]	BBBC041V1:1364 images MP-IDB:229 images	*P. falciparum* *P. vivax*	Binary	VGG-16,VGG-19, Xception, ResNet-50, customised CNN	Best performing model: VGG19 Accuracy: 99.35% F1-Score: 96.85% Sensitivity: 92.31% Specificity: 99.76% AUC: 96.03% **Cross Dataset validation:** Accuracy: 85.18% Sensitivity: 70.19% Specificity: 100% F1-Score: 84.82% AUC: 85.09%
Yang et al. [30]	2567 thin blood smear images	*P. vivax*	Binary	YOLOv2	79.22%
Krishnadas et al. [31]	MP-IDB	*P. falciparum* *P. vivax* *P. malariae* *P. ovale*	Multi-class	Yolov5 and Scaled Yolov4	Best performing model: Scaled Yolov4 Parasite classification: Accuracy: 83%
Sukumarran et al. [32]	MP-IDB and Malaria Research Centre, Unimas Sarawak	*P. falciparum* *P. vivax* *P. malariae* *P. ovale* *P. knowlesi*	Binary	YOLOv4, Faster R-CNN, SSD-300	Best performing model: YOLOv4 mAP: 93.87% Cross dataset: mAP: 84.04%

*BBBC* Broad Bioimage Benchmark Collection,* CNN* convolutional neural network, *MP-IDB* Malaria Parasite Image Database, *NLM *National Library of Medicine,* YOLO* You Only Look Once object detection algorithm

To learn and discern between the traits of malaria-infected and -uninfected RBCs, CNN models can only be trained using single-cell images of these two cell types. Segmented single-cell images from the complete thin blood smear images must be prepared to train the CNN models. Owing to the difficulties in achieving cell segmentation, a large proportion of the single-cell images used to train CNN models are those included in single-cell image datasets that are publicly available [18–26]. Numerous research makes extensive use of single-cell images, such as the 27,558 images of* Plasmodium falciparum* species from the National Library of Medicine (NLM) collection [18–26].

**Fig. 1:**
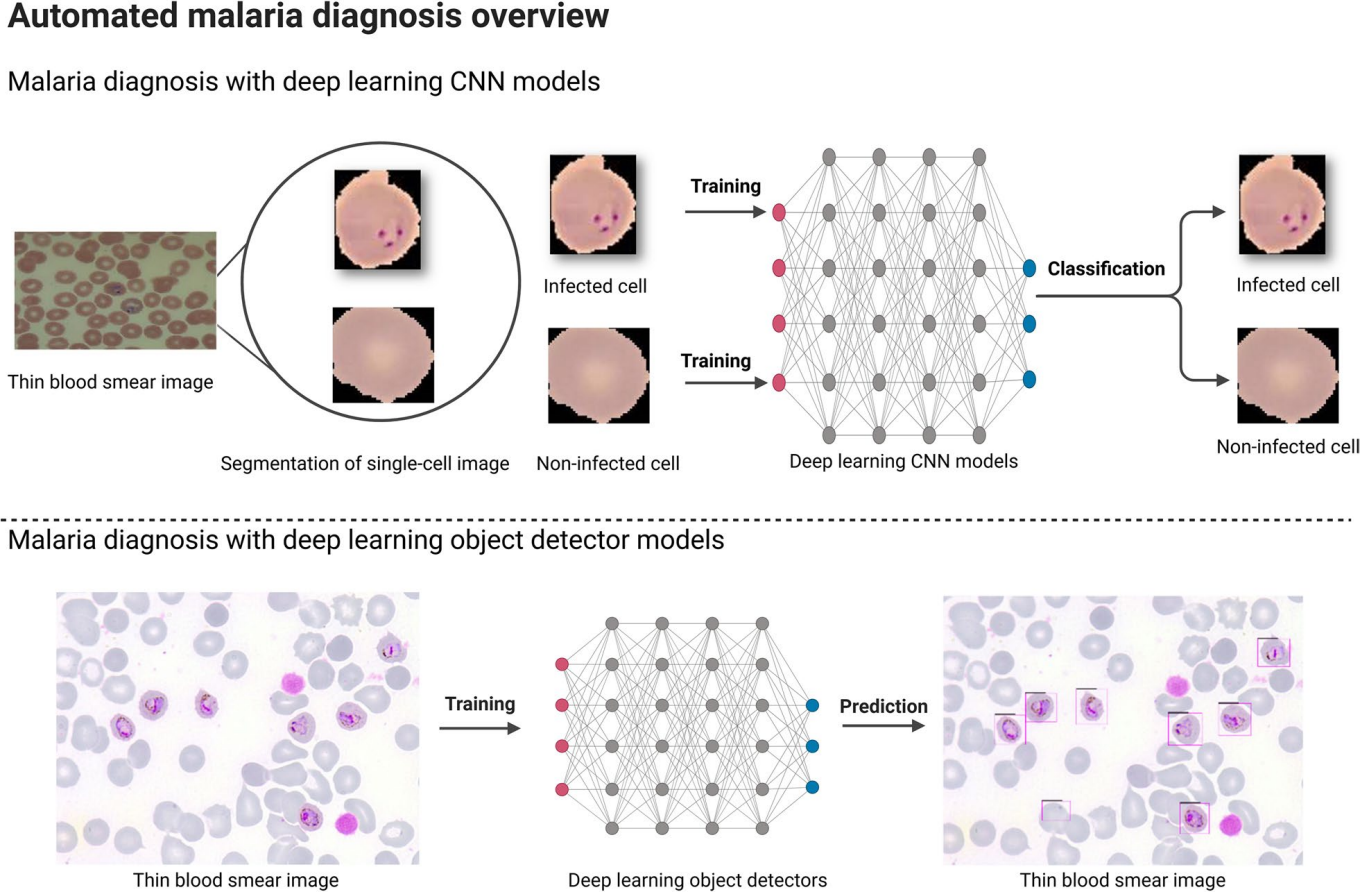
Comparison of malaria diagnosis using deep learning CNN models and deep learning object detectors. CNN, Convolutional neural network

Based on the existing literature, CNN classifiers have predominantly played a central role in the identification and classification of infected cell images. These classifiers only require the relevant images of the objects of interest for the network to understand their distinguishing characteristics. However, it is important to note from the research that automated malaria diagnosis based on single-cell images is not useful in actual clinical situations. Only single-cell image categorisation is possible with CNN models developed in earlier research (Fig. 1). Nevertheless, the infected cells coexist with other blood cells in an entire thin blood smear image. Thus, a more efficient method of immediately identifying the infected cells directly from the whole thin blood smear images is required.

### Application of deep learning object detectors in malaria diagnosis

An alternative methodology becomes available when object detection algorithms are available. The task of object detection extends beyond simple classification as it encompasses the identification of items, the assignment of labels and the accurate localisation of these objects within an image. The utilisation of CNN-based object detectors eliminates the requirement for individual cell images. Alternatively, the detectors can undergo training by delineating bounding boxes around the cells or characteristics of interest present in a complete blood smear image. The adoption of this approach places the primary emphasis on acquiring knowledge about the characteristics contained within these bounding boxes while disregarding extraneous background details. During prediction, object detectors based on CNNs effectively ascertain an object's precise location and category by accurately positioning bounding boxes directly onto the image [34, 35].

In recent years, significant progress has been made in the field of deep learning object detectors, showcasing considerable promise in the automated localisation of cells of interest in various types of blood smear images, including entire malaria thin blood smear images [30–33]. Among the studies that employed deep learning object detectors on thin blood smear images, Yang et al. [30] reported that the YOLOv2 (You Only Look Once v. 2) model achieved an accuracy of 79.22% in detecting the *P. vivax*-infected cell. In a study conducted by Krishnadas et al. [31], scaled YOLOv4 and YOLOv5 models were used for malaria parasite classification, with the models achieving an accuracy of 83% and 78.5%, respectively. In their study, Sukumarran et al. [32] compared the performances of various object detectors (YOLOv4, Faster R-CNN and SSD-300) for the detection of infected cells and found that the YOLOv4 model outperformed the other object detectors, achieving an accuracy of 93.87%. In addition, these authors tested the model’s generalisation with an independent dataset and obtained an accuracy of 84.04% [32]. The model could, however, be improved for a more lightweight and improved generalisation towards an independent dataset. To our knowledge, Sukumarran et al.'s study [32] is the only study to date that included images infected with *P. knowlesi.* While there have been a limited number of studies that have applied object detectors on malaria blood smear images, recent research has shown a potentially increasing interest in their use due to their many advantages.

### Limitations of previous studies on the application of deep learning in malaria diagnosis

Despite the widespread research that has been carried out on the application of deep learning for malaria detection, the studies performed to date are characterised by certain drawbacks. It is well known that most of the research focused on the diagnosis of malaria using CNN models (Table 1). Nevertheless, the primary drawback of CNN models is the need for single-cell pictures for testing and training. This need for single-cell imaging is a major drawback of using deep learning for diagnosing malaria (Fig. 1). To learn the properties of the object, the CNN models simply require the images that are of interest. Ultimately, using CNN models to diagnose malaria in real-time is not practical. The process of diagnosing malaria in real time involves identifying the infected cells from the entire thin blood smear image, where the infected cells coexist alongside healthy RBCs and other blood components. Therefore, in the practical context, CNN models are not relevant for automatic malaria diagnosis that entails the identification of infected cells, even if they have demonstrated promising results in classifying single cells as infected and non-infected.

In addition, cell images from various malaria parasitic infections are not used in CNN model applications. Images infected with *Plasmodium vivax* and *P. falciparum* are typically utilised extensively. Furthermore, there is a deficiency in cross-dataset validation, which involves validating the model's performances on datasets from several domains. The robustness of models on images infected by various malaria parasite species and domains is eventually called into question by these validations. Studies that have carried out cross-dataset validation [25, 29] demonstrate how important it is to examine the generalisation of the model on a separate dataset.

Deep learning object detectors can be used as a substitute to overcome the main limitation of CNN models to directly identify the infected cells from the entire thin blood smear image [30–33]. Deep learning object detectors do not require single-cell images since they can directly learn the features of an object of interest from an entire blood smear image (Fig. 1). In a similar context, they can identify the infected cells and carry out direct detection from the entire thin blood smear images.

Despite this benefit, detecting malaria-infected cells from complete thin blood smear images has not yet been extensively applied using deep learning detectors (Table 1). Two of the studies reported in Table 1 ([30, 32])focused on employing deep learning object detectors to identify the infected cells from thin blood smear images. Notably, despite being trained and tested on images that included all of the malaria parasite species, the YOLOv4 model [32] was able to identify the infected cells with an accuracy of 93.87%. Nevertheless, the YOLOv2 model [30] only recognises *P. vivax*-infected cells and has a comparatively lower accuracy. These findings indicate that entire thin blood smear images may be a valuable source of information for diagnosing malaria with the YOLOv4 model. Nonetheless, it is evident from the reduced accuracy revealed following the cross-dataset validation carried out in [32] that model modifications can strengthen the model's generalisation and optimisation.

Using object detectors to automatically identify infected cells from entire thin blood smear images replicates the current gold standard of malaria diagnosis performed by medical professionals while providing the additional benefits of a diagnosis that is more reliable, faster and less dependent on physician availability. These attributes makes it evident that object detectors have considerable promise for the automatic, real-time detection of malaria.

### Modification of YOLO models

The YOLO object detection algorithm is one of the most well-known object detectors to localise and classify objects of interest. In 2020, YOLOv4 was introduced as an object detection algorithm that outperforms alternative detectors in terms of speed and accuracy [35]. YOLOv4 has been tested in various fields, including medical images [38, 39]. Previous studies have modified and optimised the original YOLO architecture to perform better on specific datasets [34]. Computing YOLOv4 on high-resolution, thin blood smear images with a large dataset can be computationally intensive and require more powerful hardware and longer training periods to obtain satisfactory results [41]. Once trained, the model can be applied to new images to detect malaria cells. However, due to YOLOv4's complexity, the inference procedure can be computationally expensive when large thin blood smear images are processed, which can limit the automatic malaria diagnosis. To improve the model's accuracy level and robustness, the YOLO model is often further modified to handle specific problems, such as the number of model parameters, the model's size and the inference speed, despite it satisfying the classification and detection problem in many studies.

The YOLO model has undergone various modifications, such as channel pruning of YOLOv3 [46, 47] and YOLOv4 [40, 44]. This modification involved adding a constraint to the channels, assessing the importance of the constraints and removing non-critical channels without significant performance loss [44]. Channel pruning typically starts with training the YOLO model, followed by sparsity training to identify and remove channels with a lower contribution and finally by fine-tuning of the pruned model. In some cases, pruning improves detection accuracy [40], while in others, it reduces it [43, 47]. Although pruned models achieve slightly lower accuracy than the original YOLO model, they offer other benefits, such as smaller size and faster detection speed, which make these models more suitable for device deployment. Therefore, the advantages of pruned models outweigh their decreased accuracy.

In addition to channel pruning, layer pruning in YOLO models is mainly achieved by removing redundant residual blocks [47] or shortcut structures [48] from the convolutional layers of the backbone. The layers removed are specifically those that have not learned helpful information and their removal will not significantly degrade the model's performance. In a model with residual networks, such as in YOLO, the pruning can be done by removing the residual blocks from the backbone's Res-block bodies (C1-C5). Based on previous research on pruning networks with residual blocks [49, 50], removing the blocks will not have a major negative impact on the performance of the models. The residual blocks have shortcuts or skip connections that preserve essential information; therefore, removing certain layers from the network would significantly influence the model's performance. Unlike a plain network with only a single path of input to output, the residual network is a collection of many paths; therefore, removing certain paths or layers still leaves the other half path valid [51]. In addition to pruning techniques, in some studies, the backbones of the YOLO models are replaced with lighter convolutional networks, such as EfficientNet [52], MobileNetv3 [53], GhostNet [54] and DenseNet [55]. Previous research has shown that channel and layer pruning are mainly done to realise more feasible automatic detections.

The prevalence of malaria is notable in many regions that is typified by the limited availability of sophisticated computing equipment. The utilisation of lightweight models, which are obtained by means of pruning, has the potential to realise a significant transformation in the field of malaria diagnosis due to the ability of these models to facilitate the implementation of precise, efficient automatic detection on portable devices and at the point of care. Adaptability is especially advantageous in remote regions, where the prompt and dependable identification of diseases is crucial for successfully implementing both treatment and containment strategies. The utilisation of lightweight YOLO models holds significant potential to mitigating the worldwide impact of malaria since it enables the provision of sophisticated diagnostic capabilities in even the most demanding and resource-limited settings.

### Research gaps in the application of object detectors for malaria diagnosis from thin blood smear images

In previous studies, the YOLO model was used to detect both the presence of parasites and infected cells in thick [33, 62] and thin blood smear images [30–32]. Subsequently, the YOLOv3 and YOLOv4 models were modified [33, 62] to detect parasites more efficiently from thick blood smear images. However, modifications to the YOLO model to achieve a more efficient detection of infected cells in thin blood smear images have yet to be explored extensively.

The authors of one study [30] proposed a cascaded YOLOv2 model and transferred AlexNet to perform predictions of infected cells with a mean average precision (mAP) of 79.22% from *P. vivax* thin blood smear images. The unimproved YOLOv2 and improved YOLOv2 models achieved an accuracy of 71.34% and 79.22%, respectively. Although the cascaded YOLOv2 does achieve a better prediction accuracy than the YOLOv3 model, there may still be room for improvement as the model is only detecting cells infected by *P. vivax* and from the same dataset. Therefore, there is no guarantee of the model’s performance on other malarial parasitic infections and an unseen dataset. So far, to our knowledge, this study [30] is the only one to have employed the YOLO model on thin blood smear images for infected malarial cell detection. Yet the images are limited to a single species, and no cross-dataset validation was performed on the model. Based on the results of this study [30], we can deduce that other object detectors besides YOLOv2 could potentially be employed for better infected cell prediction accuracy on thin blood smear images. Only a limited number of studies have employed the YOLO model on thin blood smear images compared to the number employed on thick blood smear images (Table 2), and the former were not extensive.

**Table 2:** Performance comparison of the proposed YOLO model with those reported other published works

Authors	Techniques	Datasets/Blood smears	*Plasmodium* parasites	mAP (%)
Yang F et al. [30]	Modified YOLOv2	Self-collected 2567 thin blood smear images	*P. vivax*	79.22
Koirala. et al. [33]	Modified YOLOv3 and YOLOV4 Tiny	Self-collected 3885 thick blood smear images	*P. falciparum*	94.07
Sukumarran et al. [32]	YOLOv4	MP-IDB and 236 images from MRC-UNIMAS Sarawak	*P. falciparum* *P. vivax* *P. ovale* *P. malariae* *P. knowlesi*	84.04
Abdurahman et al. [62]	Modified YOLOv3 and YOLOv4	Publicly available 1182 thick blood smear images	*P. falcipraum*	89.73
Present study (YOLOv4-RC3_4)	Modified YOLOv4	MP-IDB The malaria parasite image database and new dataset from MRC-UNIMAS Sarawak	*P. falciparum* *P. vivax* *P. ovale* *P. malariae* *P. knowlesi*	90.07

*mAP* Mean average precision, * MP-IDB* Malaria Parasite Image Database,* MRC-UNIMAS* Malaria Research Centre-Universiti Malaysia Sarawak, * YOLO* You Only Look Once object detection algorithm

In the study of Koirala et al. [31], thin blood smear images obtained from the Malaria Parasite Image Database (MP-IDB) were utilised for a multi-classification problem, namely to classify the individual malaria parasite species using the scaled YOLOv4 and YOLOv5 models. This is the only study that we are aware of which classifies malaria parasite species using an object detector [31], but while that authors used the models to classify species on thin blood smear images, it is noteworthy that the study did not include cross-dataset validation or additional model tuning or modification to enhance classification performance. Furthermore, it is still unclear if using deep learning object detectors is ideal for performing multi-class classification of the cell according to the malaria parasite species, as there is no comparison with CNN models in terms of similar classification problems.

In the study presented here, although we used the same dataset as Koirala et al. [31], the classification problem and method of conducting the study differ. Motivated by the scenarios discussed above, our aim was to simplify YOLOv4’s performance in identifying malaria-infected cells in thin blood smear images. As compared to previous work, to the best of our knowledge, our present first study is the first to have modified the YOLOv4 object detector for malaria diagnosis from thin blood smear images.

Our ultimate aim is to train, optimise and modify the model such that it can only identify malaria-infected cells from all species as infected cells, regardless of the morphological variations. Our goal is to obtain a lighter model with faster inference time without sacrificing accuracy. We will remove the residual blocks that do not contribute to the performance of the model while maintaining high prediction accuracy. Then, we will be able to analyse and verify the importance of the various Res-block bodies and remove the layers that do not contribute sufficiently to the model’s performance. By using object detectors, we eliminate the need to prepare single-cell images.

**Fig. 2:**
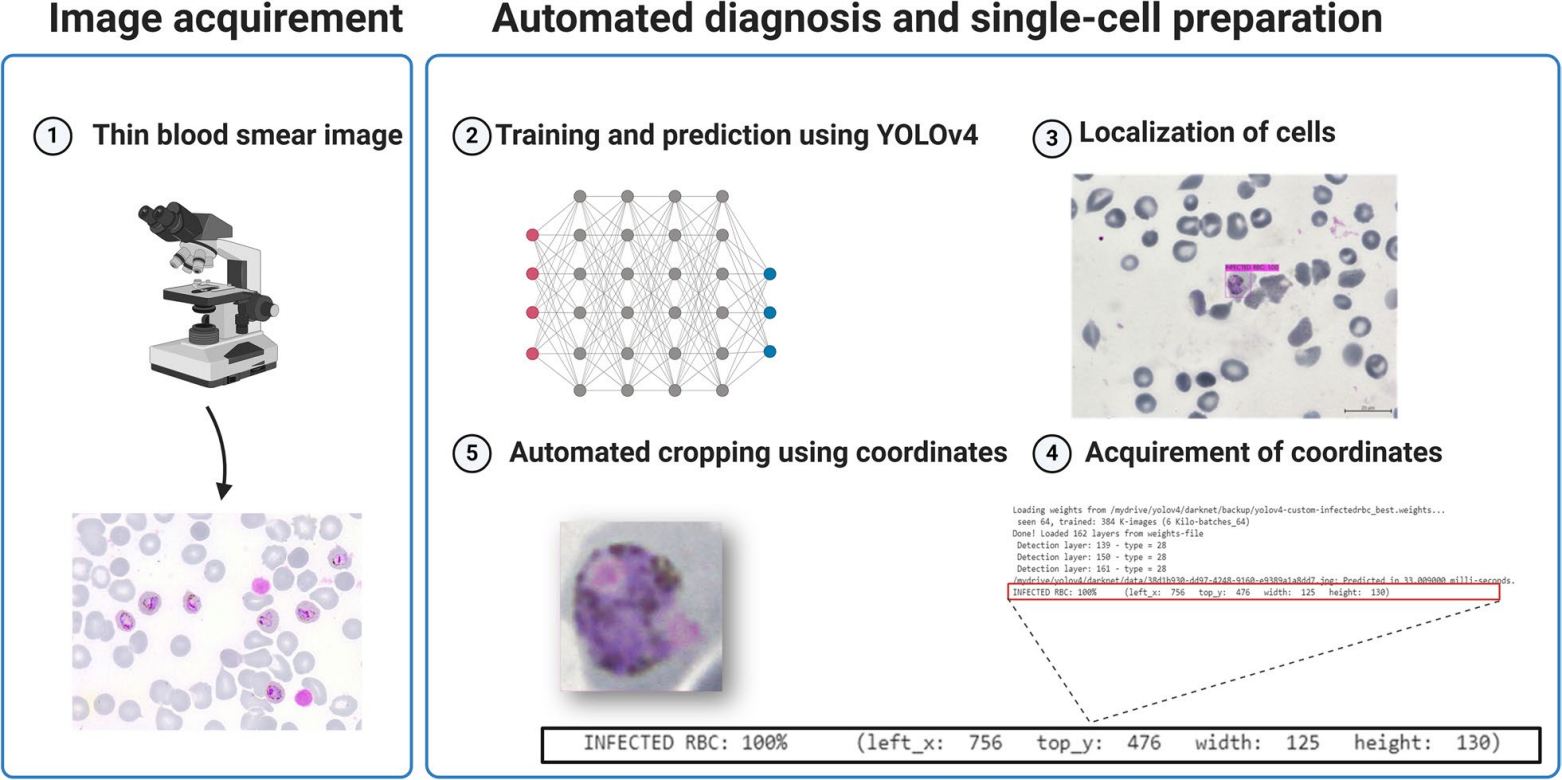
Cropping of infected cells using the coordinates of predictions by the object detectors. RBC, Red blood cell; YOLO, You Only Look Once (model)

In order to examine the impact of diverse staining data on the models, the models will also be validated using images that differ from the training images. Unlike previous research [30, 31], this investigation will also make use of *P. knowlesi*-infected pictures. As a result, we will use the bounding box coordinates (left_x, top_y, width and height) produced by the YOLOv4 model to conduct cropping of infected cells (Fig. 2). In the future, this work can be carried out by transferring these single-cell images to another classifier in order to classify them based on the species or stage of infection.

This following text is divided into the Methods, Results and Discussion sections. In the Methods section, the dataset used for this study is discussed, followed by an overview of the architecture of the YOLO4 model and the optimisation and modification techniques performed on the original YOLOv4 model. The modifications done are through layer pruning and backbone replacement. The Discussion section included comparisons of the models, comparisons with results reported in related papers and novelties of the research.

## Methods

### The proposed framework

This study is a follow-up to a previous study conducted by the authors [32]. In the previous study, the authors focused on comparing the performances of various deep learning models, such as YOLOv4, Faster R-CNN and SSD-300, for identifying malaria-infected cells, ultimately identifying the YOLOv4 model as the best-performing model among the other object detectors for malaria-infected cell prediction. Also in the previous study, no modifications of models were implemented, and all models were tested in their original version. In contrast, the technical approach in the present study is to modify the original YOLOv4 model and achieve an optimised, generalised, lightweight, rapid and accurate detection of malaria-infected RBCs from thin blood smear images. This study was performed in three main steps: (i) the training of the original Yolov4 model; (ii) layer pruning; and (iii) backbone replacement. First, we trained the original YOLOv4 model and optimised it for the localisation of malaria-infected cells using various hyperparameters. Upon obtaining the performance metrics and parameters of the original model, we then performed layer pruning on the backbone’s the C3, C4 and C5 Res-block bodies, respectively. The pruned models were then trained on the same datasets as the used in the previous training. Training and testing of all models were conducted on the same set of images. In addition to layer pruning, we replaced the CSPDarknet53 backbone with a shallower ResNet-50 backbone and trained the model with the mish and leaky ReLu activation functions. Both layer pruning and replacement with a shallower backbone allow simplification of the YOLOv4 architecture, while layer pruning also successfully achieves a reduction in model depth while maintaining the accuracy of the detection task.

### Data preparation

The images of interest in the present study are thin blood smear images. Malaria parasite species induce alterations in the morphological characteristics of erythrocytes. The* Plasmodium* species responsible for causing malaria have an impact on the morphological characteristics of the infected RBCs, with cellular morphologies varying based on the parasite species and the stage of infection. Differentiation can only be perceived in visual representations of meticulously prepared blood smears. In order to ensure the generalisability of our models, it is imperative that they possess the ability to accurately identify and localise infected cells while avoiding any limitations associated with specific species or infection phases. The YOLOv4 models, both the original and updated versions, underwent testing on two distinct datasets, as presented in Table 3.

**Table 3:** Data distribution for training and testing the model

Database	Total number of images before augmentation	Training images		Number of testing images^a^
		Number of training images before augmentation	Number of training images after augmentation	
MP-IDB (Dataset A)	210	168 (80%)	1000	42 (20%)
Malaria Research Centre, UNIMAS (Dataset B)	472	–	–	472

* MP-IDB* Malaria Parasite Image Database,* UNIMAS* Universiti Malaysia Sarawak

^a^No augmentation was performed on testing images

#### Dataset A

The first set of thin blood smear images was acquired from the publicly available dataset, MP-IDB (https://github.com/andrealoddo/MP-IDB-The-Malaria-Parasite-Image-Database-for-Image-Processing-and-Analysis). This dataset consists of 210 images of malaria infections from all malaria parasites and various stages of infection. Ground truth on the location of the infected red blood cells in every image is provided in the form of binary images. In addition, the images are classified according to species and stages of infection for better clarity in the dataset. Every image in MP-IDB is saved in JPG format, with a resolution of 2592 × 1944 pixels and a color depth of 24 bits, with a total file size of about 717 MB. This data collection was obtained solely from thin blood smears stained with Giemsa, as discussed by Loddo et al. [25]. These data were used to perform the models’ training (80%) and testing (20%). The training images were augmented to 1000 images before training using geometric augmentation techniques such as zoom-in zoom-out, flip left–right and flip top–bottom rotate (90 °, 180 °), all at various probabilities.

#### Dataset B

Additionally, as described in previous studies [58–62], archived thin blood films of malaria infections were obtained from the Malaria Research Centre, UNIMAS, Sarawak (MRC-UNIMAS). In the second dataset, referred to as Dataset B, 472 thin blood smear images were utilised. These thin blood films were fixed using absolute methanol (BDH Chemicals, London, UK) for 10 s and air-dried at room temperature. Next, the films were subjected to staining using a 10% Giemsa solution (BDH Chemicals) in Gurr® buffered water with a pH of 7.2 (BDH Chemicals) for 30 min, as recommended by the protocol outlined by the WHO [2]. All thin blood films were examined using a microscope (model BX53; Olympus Corp., Tokyo, Japan) at a total magnification of 1000× with immersion oil. Images were captured using a digital camera (model DP25; Olympus Corp.) mounted to the microscope and analysed using Digital Imaging Solution Cell B software (Olympus Corp.). This dataset was compiled in a manner akin to Dataset A, encompassing multiple* Plasmodium* species of human malaria parasites, namely *P. falciparum, P. malariae, P. vivax,* and *P. ovale*, along with the inclusion of *P. knowlesi* images. The inclusion of this dataset is for cross-validation purposes and to ensure thorough assessment of the performance, robustness and generalisation capabilities of the models under examination.

### YOLOv4 architecture

**Fig. 3:**
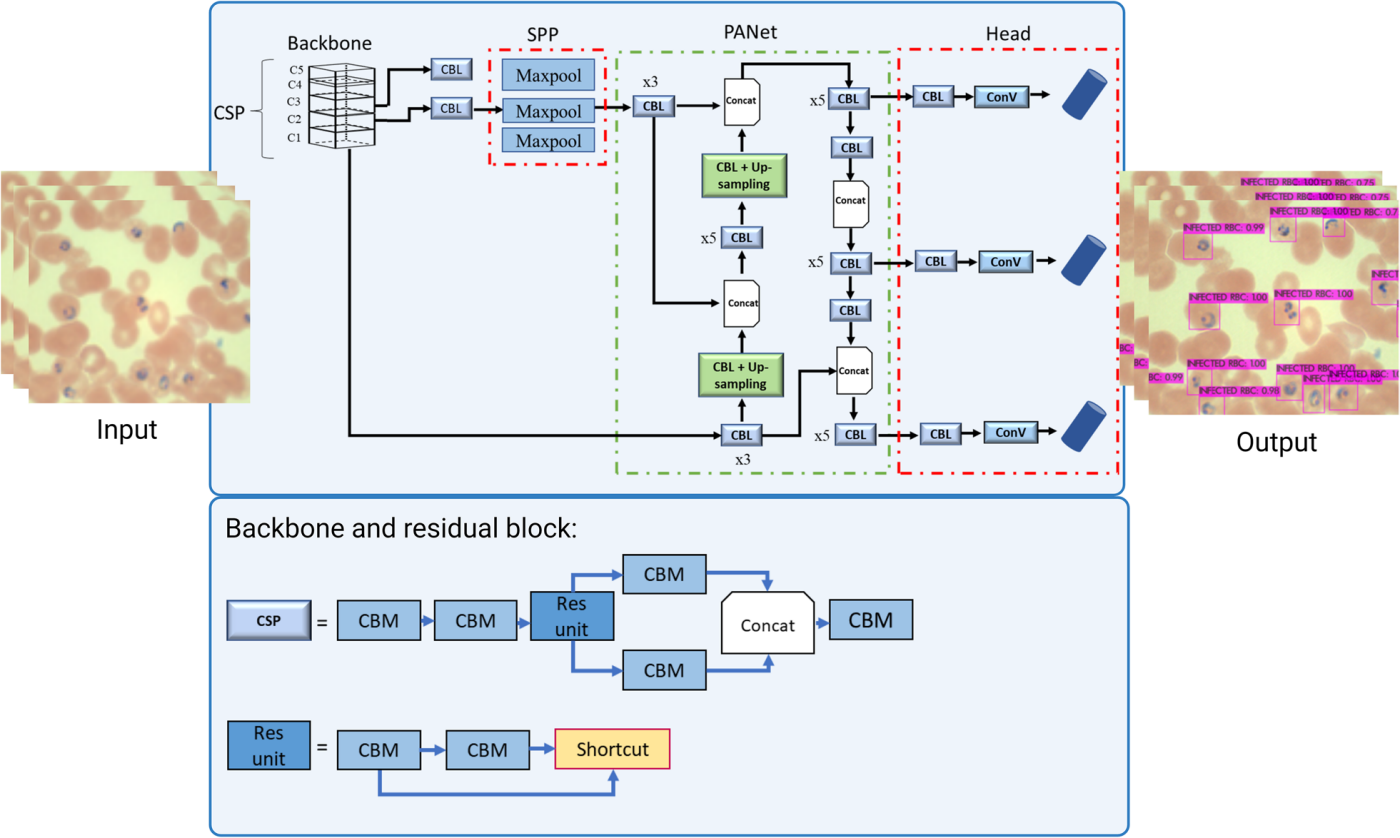
Network structure of YOLOv4. CSP, cross-spatial connection; SPP, spatial pyramid pooling layer; PANet, Path Aggregation Network; CBM, Convolutional, Batch Normalisation, and Activation; CBL, Convolutional, Batch normalisation, and Leaky-ReLU; Conv, convolutional; Concat, concatenation

YOLOv4 is a one-stage object detector that treats object detection as a simple regression problem. As shown in Fig. 3, In YOLOv4, when an input image is fed, the network gives the class probabilities and classifications with the bounding boxes on the localised objects in a single pass. Unlike two-stage object detectors as the Faster Region-based Convolutional Neural Network (Faster R-CNN), YOLOv4 performs prediction with a single fully connected layer. The model can be optimised from end to end since the detection pipeline is just one network. The network structure of the YOLOv4 model is shown in Table 4. The model has three main parts: the backbone, neck, and head [35].

**Table 4:** Network structure of YOLOv4. Network structure of the YOLOv4 model

YOLOv4 mels	Number of layers	Type
CSPDarkNet53 without the fully connected layer	0	CBL
	1–7	**Res**
	8	Conv
	9	Route
	10–20	**2Res**
	21	Conv
	22	Route
	23–51	**8Res**
	52	Conv
	53	Route
	54–82	**8Res**
	83	Conv
	84	Route
	85–101	**4Res**
	102	Conv
	103	Route
Feature fusion layer and output layer	104–107	4CBL
	108–113	SPP
	114–117	4CBL
	118	Up-sample
	119	Route
	120	Conv
	121	Route
	122–127	6CBL
	128	Up-sample
	129	Route
	130	CBL
	131	Route
	132–137	6CBL
	138–139	CBL + YOLO
	140	Route
	141	CBL
	142	Route
	143–148	6CBL
	149–150	Conv + YOLO
	151	Route
	152	CBL
	153	Route
	154–159	6CBL
	160–161	Conv + YOLO

Head: The main function is to locate the bounding boxes and classify the objects of interest. The coordinates and the scores of every bounding box are generated

*YOLO* You Only Look Once (model), *CBL* Convolutional, Batch normalisation, and Leaky-ReLU (Feature extractor), *Res* residual block

#### Backbone

The backbone comprises three main parts: the bag of freebies, the bag of specials and the CSPDarkNet53 network. The bag of freebies increases the model's robustness by performing data augmentations to increase the variability of the training images. The bag of specials is the post-processing module used in the YOLOv4 model to improve the accuracy with a slight increase in the inference cost. Examples of the modules are the mish activation functions and cross-stage partial connections (CSP) for the backbones. The backbone has 53 layers and five residual bodies (C1-C5) for extracting deep features. Unlike YOLOv3, YOLOv4 utilises the cross-spatial connection into the Darknet-53 backbone for feature extraction. The CSP is derived from the Densenet network, which uses the previous input and concatenates it with the current input before moving to the dense layer [35].

#### Neck

The neck is part of the system that concatenates the feature maps from the bottom-up and top-down stream before feeding into the head for object detection. It mainly consists of the Spatial Pyramid Pooling (SPP) layer and PANet. The SPP block increases the receptive field by separating the most significant features from the backbone to the head of the model, thereby changing the convolutional features of different sizes into pooled features with the same length [35], whereas the PANet performs parameter aggregation from different levels of backbones [35]. The CBL (Convolutional, Batch normalisation, and Leaky-ReLU) is used for feature extraction in the neck. The difference between the CBL and CBM (Convolutional, Batch normalisation and Mish) modules is the activation functions used.

### Residual blocks in the YOLOv4 model

**Fig. 4:**
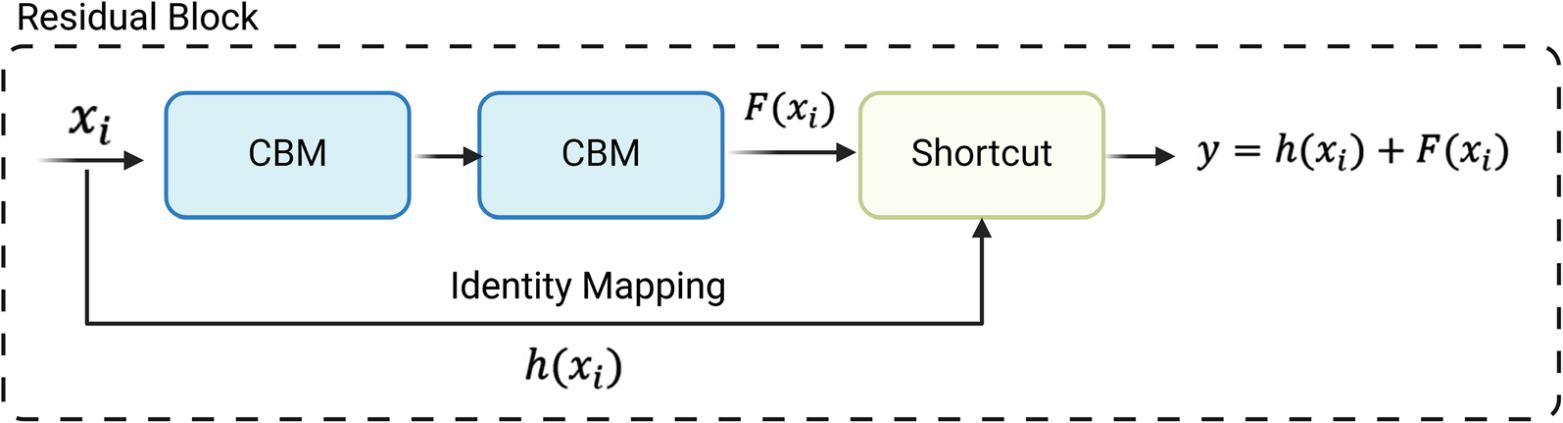
Building blocks of the residual learning module. CBM, Convolutional, Batch normalisation and Mish (modules)

The aim of the present study was to simplify the complexity of the current model by utilising a technique called layer pruning. The removal of shortcut structures from the C3, C4 and C5 (C3-C5) residual bodies was explored in a previous study [48]. Our present study specifically investigates the pruning of redundant residual blocks and their related shortcut structures inside the Res-block bodies C3-C5 of the underlying network architecture (Fig. 4). The residual blocks are essential components that are distinguished by their dual CBM modules and a corresponding shortcut layer. In networks that incorporate residual blocks, the transfer of characteristics from lower levels to higher layers is inherent [57]. Nevertheless, within the domain of deep learning, it is widely acknowledged that when neural networks grow in depth, a critical juncture exists where the accuracy of the model reaches a plateau and may even experience a sudden decline. This phenomenon cannot be solely attributed to overfitting [57]. Therefore, to solve this degradation problem, a deep residual network was introduced. This approach adds a shortcut or skip connection within the convolutional layers. With shortcuts, identity mapping is performed, and their outputs are added to the inputs of the deeper stacked layers. The model learns the residual functions with the shortcut connection, and all the features that the deeper layers learn from the shallower layer are denoted as:

$$y=h({x}_{i})+ \sum F({x}_{i})$$

where *y* = output, $h({x}_{i})$ is the identity mapping and $F({x}_{i}, )$ is residual mapping to be learned.

An identity connection *h(x)* comes from the input *x*; the layers try to learn the residual, *F(x)* (Fig. 4). In short, layers in a plain network learn the true output *h*(*x*); however, layers in a residual network also learn the residual.

### YOLOv4 architecture modification by layer pruning and backbone replacement

Training was all conducted in GoogleColab with Nvidia T4 GPU. The models were all trained for 6000 epochs with a learning rate of 0.001 and an input image size of 416 × 416. The original YOLOv4 model was trained at a learning rate of 0.001 and 0.0001. The learning rate of 0.001 was chosen as the final one following comparison af all model's performances. The models were all trained and tested on the same images to avoid bias in comparing their performances. In this study, the original YOLOv4 model was first trained and optimised to detect the malaria-infected RBCs from whole thin blood smear images. Although the YOLOv4 model is able to detect infected RBCs effectively, the network structure is relatively large for our dataset and problem and caused massive calculations and B-FLOPS (billion floating-point operations). Since our aim is to reduce the model's complexity without compromising the model's accuracy, we chose to modify the architecture by conducting direct pruning of residual blocks from the C3–C5 Res-block bodies of the model. With pruning, redundant residual blocks that do not contribute much to the model's efficiency can be removed. Removing residual blocks will not drastically reduce the model’s performance as they do not depend on each other [57]. However, removing an increased number of residual blocks can significantly impair accuracy. Therefore, in this study, the pruning was conducted from the C3–C5 Res-block bodies layer to avoid over-pruning.

The YOLOv4 model has a total of 22 residual blocks in the Res-block bodies. However, we only focused on removing the residual blocks from the C3–C5 Res-block bodies, which have eight, eight and four residual blocks, respectively. The candidates of the residual blocks removed in each layer upon modification are shown in Table 5. For example, for the candidate 1 model, 'x' represents that pruning occurs on the C3 Res-Block body, whereas ‘/’ indicates that no pruning occurs on the C4 and C5 Res-block bodies. The ‘[52 × 52 × 128]’ indicates the image output sizes on the remaining layers on the C3-Res block body. Similarly, for the candidate 2 model, pruning occurs only on the C4 Res-block body. The candidate 3 and 4 models are shown with pruning on the C5 and C3-C4 Res-block bodies, respectively. Table 6 gives an overview of the number of residual blocks in the respective Res-block bodies before and after pruning. For example, model YOLOv4-RC3 indicates is shown with residual blocks removed from the C3 Res-block body (Table 6). In contrast, YOLOv4-RC4 is shown with residual blocks removed from the C4 Res-block body of the model. It should be noted that for the backbone replacement with the ResNet-50 network, the ‘L’ and ‘M’ indicate activation of the Leaky ReLu and mish function, respectively (Table 7).

**Table 5:** Network architecture of the pruned models

Candidate model	Res-block 3 (C3)	Res-block 4 (C4)	Res-block 5 (C5)
	1 × 1/1 3 × 3/1		
Candidate 1 (YOLOv4-RC3)	'x' [52 × 52 × 128]	‘/’ [26 × 26 256]	‘/’ [13 × 13 × 512]
Candidate 2 (YOLOv4-RC4)	‘/’ [52 × 52 × 128]	'x' [26 × 26 256]	‘/’ [13 × 13 × 512]
Candidate 3 (YOLOv4-RC5)	‘/’ [52 × 52 × 128]	‘/’ [52 × 52 × 128]	'x' [13 × 13 × 512]
Candidate 4 (YOLOv4-RC3_4)	'x' [52 × 52 × 128]	'x' [26 × 26 256]	‘/’ [13 × 13 × 512]
Candidate 5 (YOLOv4-RC3_5)	'x' [52 × 52 × 128]	‘/’ [26 × 26 256]	'x' [13 × 13 × 512]

'x' indicates that pruning occurs on the Res-Block bodies; ‘/’ indicates that no pruning occurs on the Res-block bodies; values in square brackets indicate the image output sizes on the remaining layers of the Res-block bodies

*YOLO* You Only Look Once (model)

**Table 6:** Residual block removal from the C3, C4 and C5 layers of the CSPDarknet53 backbone

Modifications	Models	Res-block bodies	Residual blocks (*n*)	
			Before	After
Residual blocks pruning	YOLOv4-RC3	C3	8	3
	YOLOv4-RC4	C4	8	3
	YOLOv4-RC5	C5	4	2
	YOLOv4-RC3_4	C3 and C4	8, 8	3, 3
	YOLOv4-RC3_5	C3 and C5	8, 4	3, 2

*YOLO* You Only Look Once (model)

**Table 7:** Detection of infected red blood cells on Dataset A using the original YOLOv4 and modified models

Modifications	Model	Precision (%)	Recall rate (%)	F1-score (%)	mAP (%)	Training time (h)	Inference time (per image) (ms)	B-FLOPS	Size (MB)
Original	YOLOv4	84	95	89	93.87	48	726.66	59.57	244.40
Residual block pruning	YOLOv4-RC3	84	92	88	91.65	35	678.53	47.59	242.40
	YOLOv4-RC4	83	92	87	92.84	37	703.82	51.21	233.20
	YOLOv4-RC5	85	89	87	92.47	37	704.48	57.61	222.10
	YOLOv4-RC3_4	83	89	86	88.09	32	676.18	37.35	221.50
	YOLOv4-RC3_5	77	77	77	76.56	32.5	680.01	45.64	220.4
Backbone replacement	YOLOv4- ResNet-50L	70	84	76	79.70	28	719.50	37.33	209.30
	YOLOv4-ResNet-50 M	74	86	80	81.43	28	884.82	37.33	209.30

*B-FLOPS* Billion floating point operations, *F1-score* balance between precision and recall, *mAP* mean average precision

In a residual network, the feature information is well preserved via skip connections; therefore, removing a specific layer from the model has only a minor influence and no destructive impact on the model’s performance [53]. In some models, removing the block may positively influence the model’s performance metric, whereas there is no improvement in metrics depending on the feature representations the layers contribute to. Therefore, by removing the residual blocks accordingly by Res-block bodies and with proper retraining, we can identify the layers with the best discriminative features and remove the redundant blocks that do not contribute to any notable extent to the efficiency and accuracy of the model.

In addition to pruning the redundant residual blocks, we replaced the CSPDarknet53 backbone of the model with a shallower ResNet-50 network. The model was then retrained on the same settings as the previous training. The CSPDarknet53 was chosen as the final backbone of the YOLOv4 model as it demonstrates greater ability in detector accuracy upon various improvements. However, the model has been trained with a vast dataset and many class classifications, which is different from the situation in the present study. The performance of a particular object detector changes according to the dataset and the detection problem. Similarly, the model's performance might change at a different backbone structure according to the dataset and detection problem. The backbone with ResNet-50 was replaced to identify if a shallower backbone with similar shortcuts and skip connection layers would perform better on the malaria dataset while concurrently decreasing the computation complexity.

### The performance metric for model evaluation

The model presented in this study was tested with test images from Dataset A and was cross-verified with the independent Dataset B. An independent dataset is used to test the model’s generalisation in detecting the infected cells on images with different stain images as the training dataset.Five main performance metrics were used to evaluate the models’ performances.

1 $$Precision=\frac{TP}{TP+FP} x 100\%$$

2 $$Recall=\frac{TP}{TP+FN} x 100\%$$

3 $$F1-score=\frac{2[precision\left(class=1\right) .recall\left(class=1\right)]}{precision(class=1)+recall(class=1)}$$

4 $$mAP=\frac{1}{n}{\sum }_{k=1}^{k=n}AP\left(n\right)$$

Precision (Eq. 1) calculates the correct predictions by the model, with higher precision indicating that the model has more true positive detections. Recall rate (Eq. 2) represents the intolerance of the model towards false negatives. F1-score (Eq. 3) is the balance between precision and recall, and is commonly given more importance when there is an uneven class distribution of data. Average precision (AP) is the area under the precision-recall curve. The precision-recall curve is the trade-off between the precision and recall of a model at different thresholds. A high area under the curve represents both the high recall and high precision of the model. AP is the numerical representation of the precision-recall curve, and this metric summarises the weighted mean precision for each threshold with the increase in recall. AP is calculated for each class. mAP (Eq. 4) is the mean of the AP of all the classes detected by the model. In this study, there is only one class (*k* = 1); therefore, the AP is the mAP. The mAP will be the object detectors' primary performance evaluation criterion [47]. In addition to mAP, the B-FLOPS, inference time and model size are also compared. The B-FLOPS is the number of floating-point operations that the model can perform in a second and is a measure of the model’s complexity.

## Results

### Comparisons of the original YOLOv4 model and modified models

The mAP achieved by the original YOLOv4 and the modified YOLOv4 models on the test images from Dataset A are shown in Table 7. The models are trained for 6000 epochs; the best weights are chosen as the final weights to perform predictions on the images. This was also done to avoid using very overfitted weights throughout training. From the results, the original YOLOv4 model outperforms the modified models on the test images from Dataset A, followed by the YOLOv4-RC4, YOLOv4-RC5 and YOLOv4-RC3 models which achieve > 90% accuracy with a reduced size and B-FLOPs. Although the original YOLOv4 model achieves the highest accuracy, evaluating the models on an independent dataset is necessary. Therefore, the predictions were further evaluated on independent Dataset B (Table 8). The best weights of the respective models were tested on Dataset B and, based on the results, the original model’s performance was seen to drop by > 12%. However, not only the prediction rate for the original model degraded, but also that for the modified models.

**Table 8:** Detection of infected RBC on Dataset B using the original YOLOv4 and modified models

Modifications	Model	Precision (%)	Recall rate (%)	F1-score (%)	mAP (%)	Inference time (ms)	B-FLOPS	Size (MB)
Original	YOLOv4	61	86	72	81.43	905.65	59.57	244.40
Residual Block pruning	YOLOv4-RC3	50	95	66	88.91	695.54	47.59	242.40
	YOLOv4-RC4	50	91	65	85.20	695.98	51.21	233.20
	YOLOv4-RC5	61	93	73	89.84	731.43	57.61	222.10
	YOLOv4-RC3_4	59	96	74	90.70	684.93	37.35	221.50
	YOLOv4-RC3_5	59	93	72	88.09	690.53	45.64	220.40
Backbone replacement	YOLOv4- ResNet-50L	54	83	65	76.95	892.80	37.33	209.30
	YOLOv4-ResNet-50 M	65	81	72	78.96	905.39	37.33	209.30

*B-FLOPS* Billion floating-point operations, *F1-score* balance between precision and recall, *mAP* mean average precision

Nevertheless, all of the pruned models were found to outperform the original YOLOv4 model on Dataset B. The original YOLOv4 model has possibly overfitted on the training images or the model with too many layers is too deep for our dataset and problem, causing both a higher training error and the accuracy to drop significantly on unseen data. An overfitted model starts to fit too closely to the training data. Therefore, the original model performed well on the test images that were similar to the training images from Dataset A but failed to generalise on the images of unseen Dataset B. Comparatively, the pruned models performed better on Dataset B compared to the original YOLOv4 model, suggesting that less overfitting took place on the optimised models mainly because the number of layers was reduced.

**Fig. 5:**
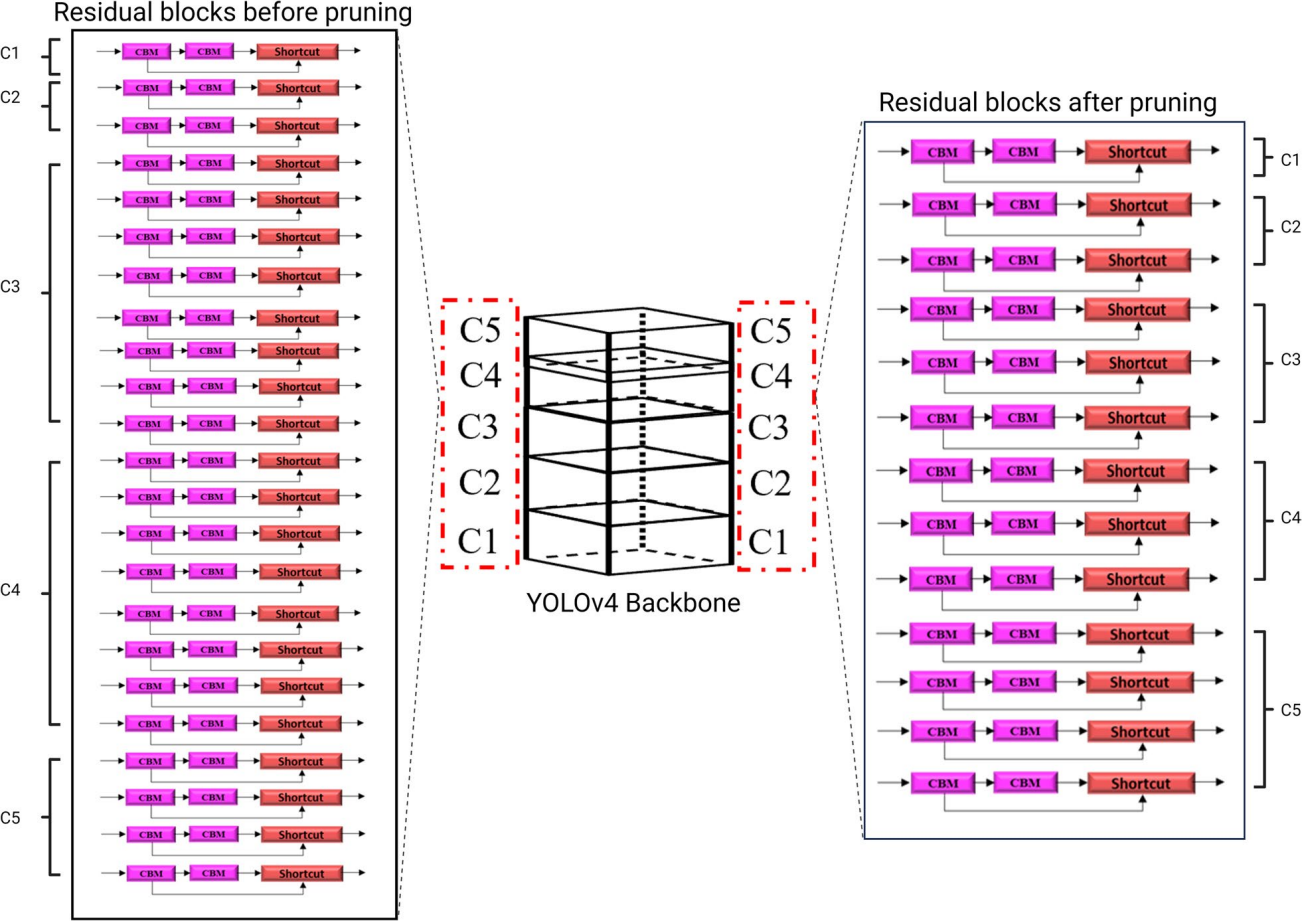
Visual representation of the removal of residual blocks from C3 and C4 Res-block body. YOLO, You Only Look Once (model)

From the analysis of results on Dataset B, the YOLOv4-RC3_4 model (Fig. 5) attained the highest detection accuracy of 90.70% for malaria-infected RBCs compared to the original YOLOv4 and other pruned models. This model saves about 22% of the B-FLOPS while achieving > 9% higher accuracy and is 23 Mb smaller than the original model. The visual representation of the YOLOv4-RC3_4 Res-block body before and after the removal of residual blocks is shown in Fig. 5. YOLOv4-RC3 and YOLOv4-RC5 followed YOLOv4-RC3_4 in terms of detection accuracy, obtaining mAP of 88.91% and 89.84%, respectively, still outperforming the YOLOv4 original model. We also compared the B-FLOPS and model size. All pruned models have less B-FLOPS and smaller model size than the original YOLOv4 model, indicating that the former models are less complex.

Regarding the number of layers, the YOLOv4-RC3 and YOLOv4-RC4 models have the same number of layers upon removal of the five residual blocks from each model. In contrast, the YOLOv4-RC5 model has more layers, as only two residual blocks are removed (Table 6). However, the size of model YOLOv4-RC3 is larger than that of the YOLOv4-RC4 and YOLOv4-RC5 models, possible because the C3 Res-block body carries fewer parameters, and there are more parameters in the backbones C4 and C5 Res-block bodies where no pruning occurred. Therefore, removing the residual blocks from C3 only slightly affects the model size. This is proven when the model size of YOLOv4-RC4 is reduced upon pruning the C4 residual blocks and further reduced in YOLOv4-RC5 upon just pruning two residual blocks from C5. Our interpretation was that the C5 carries more parameters, followed by C4 and C3 Res-block bodies.

Based on the results on Dataset B (Table 8), the YOLOv4 models with the ResNet-50 backbone did not perform well compared to the pruned models. Comparison of the pruned models with a shallower backbone revealed that the size of the models with the ResNet-50 backbone is relatively smaller than the size of the original and the pruned models, respectively. The fewer layers may contribute to this and indicate a reduction in the models' parameters. However, the detection accuracy of the pruned models was found to be relatively higher, and although the models with the ResNet-50 backbone have higher mAP than the original YOLOv4 model on Dataset B, their recall rate is relatively lower than that of the original model, leading to more false negative predictions (Table 9). Eventually, this situation is not satisfactory as the aim of study was to detect as many true positive cells as possible while reducing the model’s complexity. We interpreted this result as the YOLOv4 models with the CSP-DarkNet53 backbone performing better feature extraction than those with the ResNet-50 backbone.

**Table 9:** True positive predictions on Datasets A and B

	Dataset A	Dataset B
Model	*355*	*740*
YOLOv4	337	635
YOLOv4-RC3	327	704
YOLOv4-RC4	326	674
YOLOv4-RC5	315	685
YOLOv4-RC3_4	317	708
YOLOv4-RC3_5	272	631
YOLOv4- ResNet-50L	297	616
YOLOv4- ResNet-50 M	307	596

*YOLO* You Only Look Once (model)

**Fig. 6:**
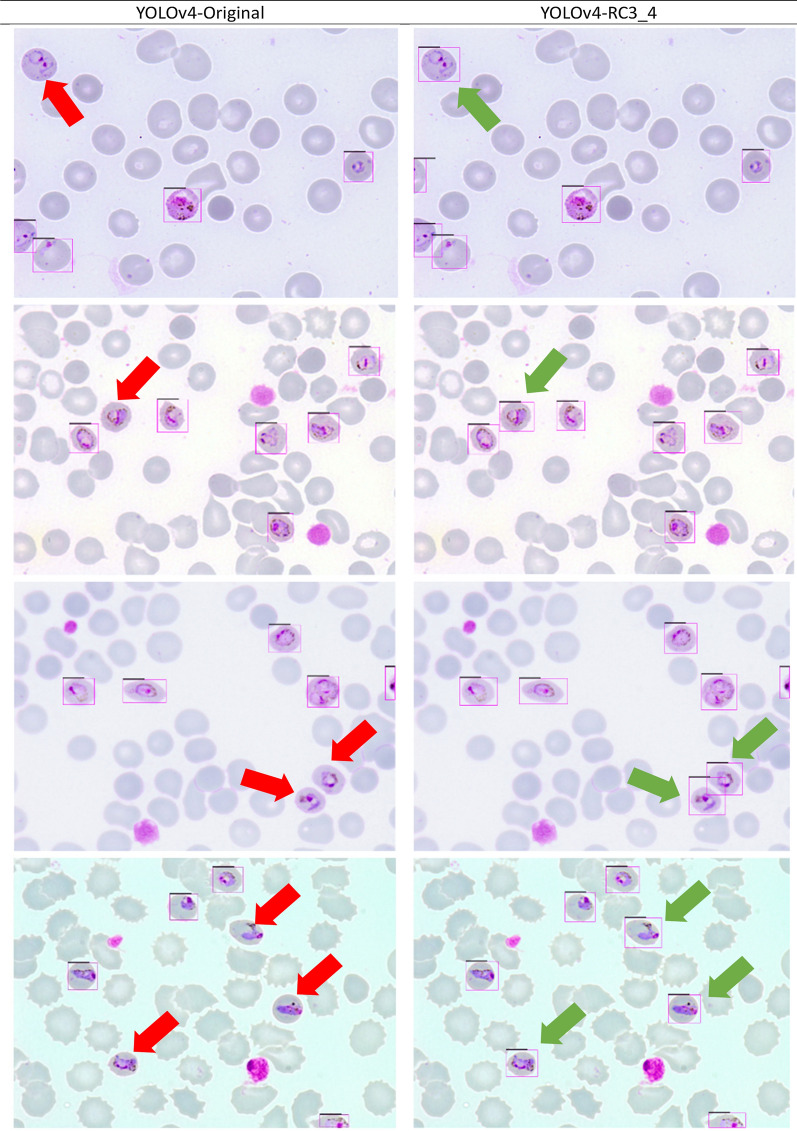
Comparison of detection performance by the original YOLOv4 model and the YOLOv4-RC3_4 model. Red arrows indicate cells not detected by the original YOLOv4 model, green arrows indicate the same cells detected by the YOLOv4-RC3_4 model. YOLO, You Only Look Once (model)

The predictions of the original YOLOv4 and best-performing YOLOv4-RC3_4 models were further evaluated on the images from Dataset B. Figure 6 shows a few of the predictions performed. The original YOLOv4 model has a lower recall rate than the YOLOv4-RC3_4 model, and this significantly impacts the performance of the original YOLOv4 model (Table 8). For further analysis, the true positive predictions of the models were compared (Table 9). The best-performing YOLOv4-RC3_4 model was found to detect 73 more infected cells than the original YOLOv4 model on Dataset B. It should be highlighted that Dataset B contains images of cell infected by *P. knowlesi,* unlike Dataset A, which only contains cells infected human-exclusive* Plasmodium* species. The prediction results of the YOLOv4-RC3_4 model show that the model can still generalise and detect malaria-infected cells based on the infected cells’ features that it has learned.

**Fig. 7:**
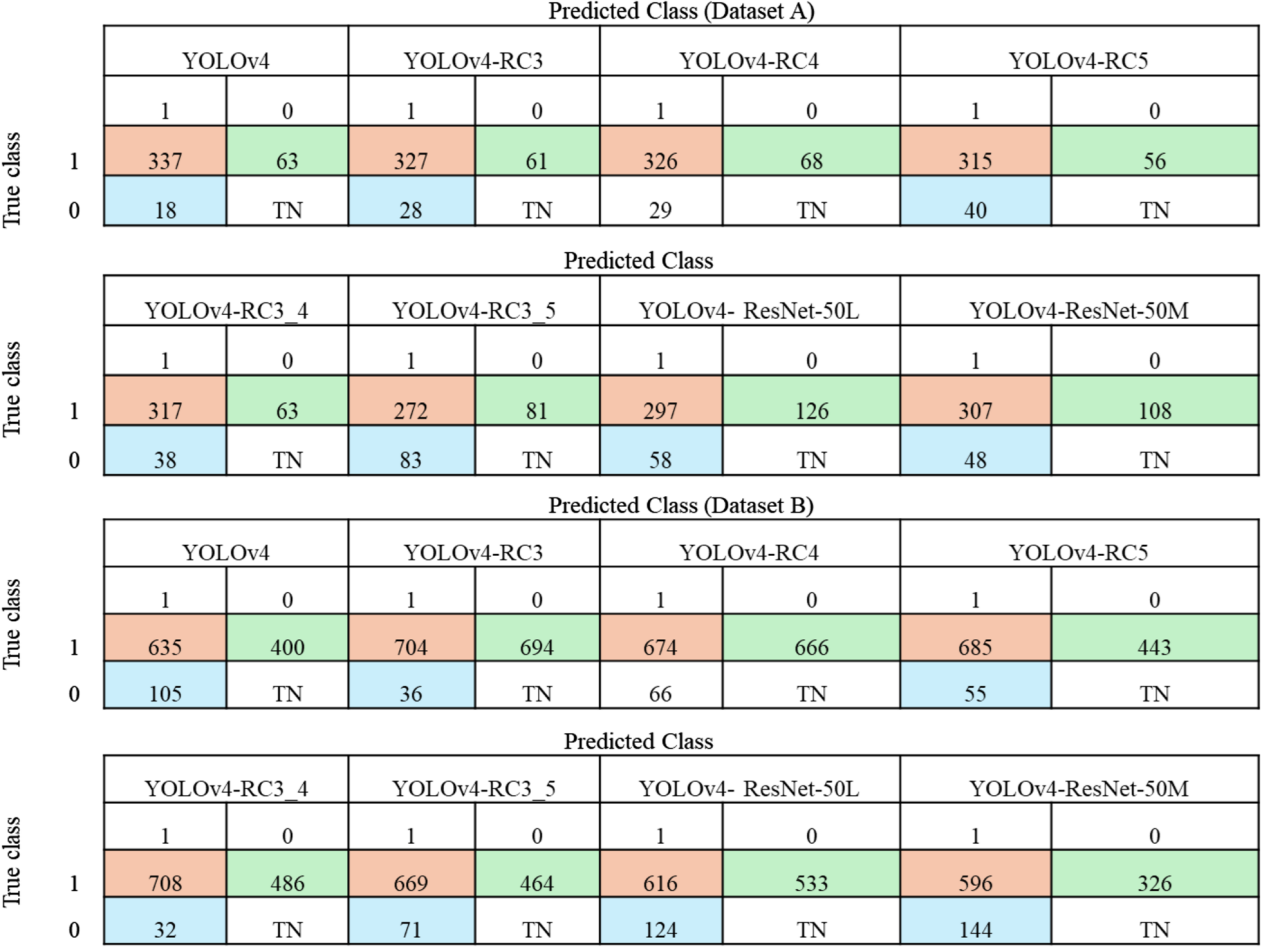
Confusion matrix of predictions by YOLOv4 models. YOLO, You Only Look Once (model)

**Fig. 8:**
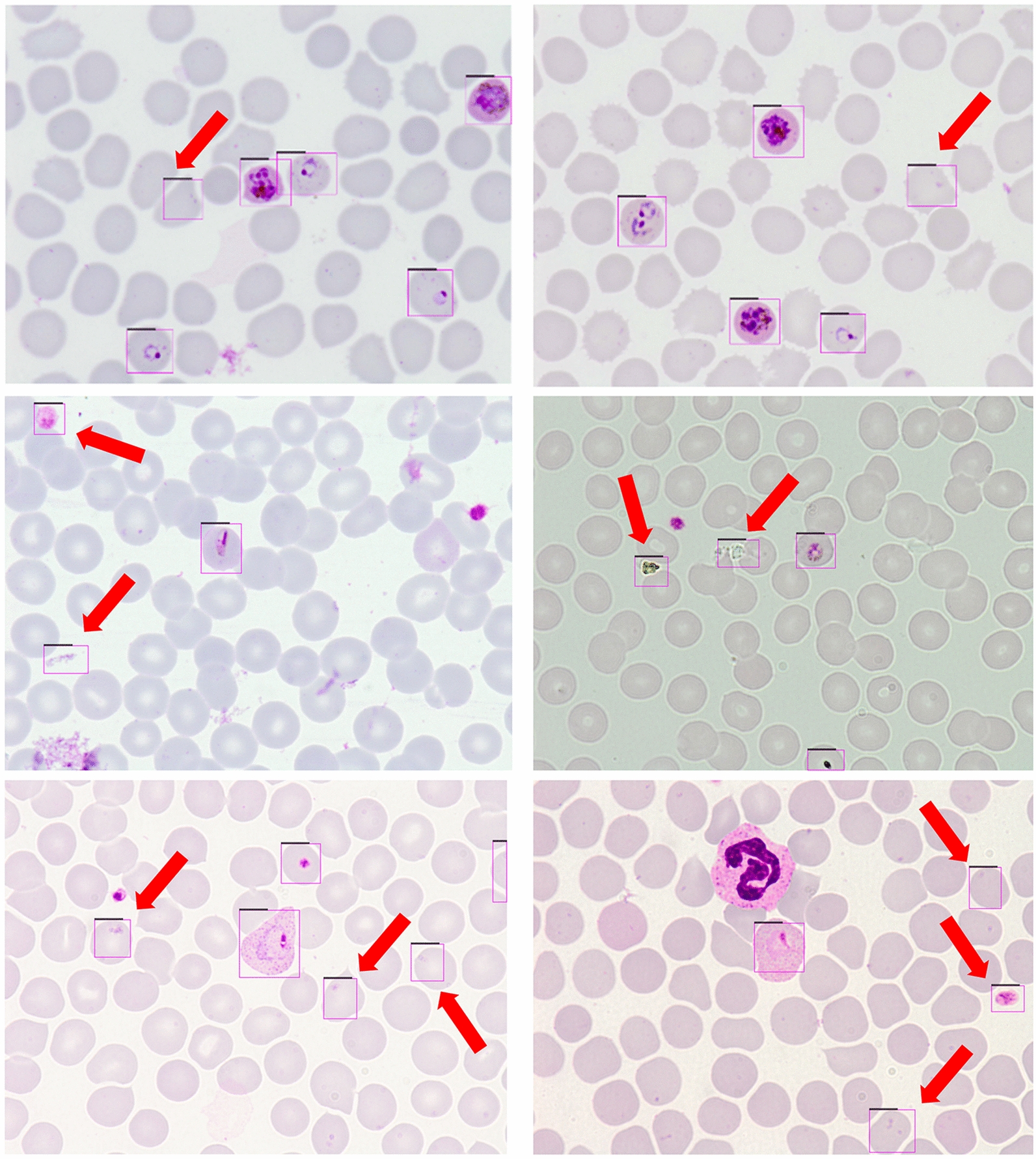
Examples of false positive predictions by the YOLOv4-RC3_4 model. YOLO, You Only Look Once (model)

Upon analysing the performance of the pruned models on the test images, we noted that the false positive predictions played a huge role in determining the models’ performances (Fig. 7). For example, the performance of the YOLOv4-RC3_4 model on the test images was found to be mainly negatively impacted by the false positive predictions with a precision of 59%. Based on Fig. 7, the YOLOv4-RC3_4 has 486 false predictions on 472 test images from Dataset B, with an average of one to two false positive predictions in an image. As shown in Fig. 8, the model specifically detects faint stains or small material as infected cells. In some images, the small material might represent the structure of ring-stage malaria-infected cells that the model could falsely recognise. However, based on the results and the understanding of performance metrics, the precision of the models typically drops when the recall rate increases. This also explains the highest precision achieved by the original YOLOv4 model and, consequently, its lowest recall rate. Although there is a trade-off between precision and recall, in this study, a model with a high recall rate is essential to flag as many malaria-infected cells as possible. The YOLOv4-RC3_4 model can attain a good recall rate and decent precision simultaneously. It is worth noting that the model cannot detect 32 infected cells, but in an image of more than two infected cells, it can still detect at least one or two infected cells, still flagging the person as infected.

## Discussion

### Research findings

Based on our study findings, we have successfully modified the YOLOv4 model for detecting malaria-infected cells on thin blood smear images. Our study highlights that pruning redundant residual blocks from the Res-block bodies can enhance the model’s accuracy, generalisation and robustness while reducing its size, B-FLOPS and inference time. To our knowledge, this is the first study to explore this method for malarial cell detection, and we believe it has significant potential for improving malaria detection.

The YOLOv4 model has undergone modifications in previous research applications to simplify the model and increase the inference time. These modifications typically involve channel [41, 44] or layer pruning [48], which removes unnecessary channels and layers that do not contribute to the model's detections or performance. Channel pruning results in a 0.2% accuracy drop, which is compensated for by other benefits, such as a smaller model size and faster inference speed [44]. Similarly, pruning shortcut layers from the C3-C5 backbone reduces the model size while improving accuracy by 2.6% [48]. These modifications demonstrate that the model's architecture can be improved without sacrificing accuracy and complexity, making it more feasible for device deployment.

In the present study, we took advantage of the residual blocks present in the backbone of the YOLOv4 model. The layer in a residual network comprises multiple convolutional layers, which are called residual blocks [51]. Previous studies on residual networks showed that the model's performance can still be preserved following the pruning of residual blocks [33, 47, 49]. In a residual network, the paths are not heavily reliant on each other and interactions can still be reconstructed to make up for performance loss caused by pruning. However, removing residual blocks beyond necessary can significantly decrease accuracy, as noted by Veit et al. [51]. Therefore, we removed the residual blocks in the YOLOv4 model only from the C3 to C5 Res-block bodies.

Our findings showed that it is possible to directly prune residual blocks to determine the importance of Res-block bodies and avoid excessive pruning. We discovered that it is more effective to prune the residual blocks of the backbone separately from the C3-C5 Res-block bodies rather than prune them simultaneously. By pruning the residual blocks separately, we can pinpoint which Res-block bodies contribute less to prediction accuracy and also prevent over-pruning. As a first step, we pruned the residual blocks from the C3 (YOLOv4-RC3); pruning of the C4 Res-block bodies followed (YOLOv4-RC4). We interpreted the results as indicating that pruning the residual blocks from the C3 (YOLOv4-RC3) and C4 (YOLOv4-RC4) Res-block bodies results in a model with the same number of layers. Both these models have a better prediction accuracy than the original YOLOv4 model, highlighting that reducing the layers can eventually improve the training and the model’s robustness. The difference in the sizes of these two models indicates that the C4 Res-block body carries more parameters and that removing them causes the size of the models to decrease significantly. At the same time, the YOLOv4-RC3 model has a smaller number of B-FLOPs, although this model has a larger size than the YOLOv4-RC4 model. This observation indicates that C3 Res-block bodies compute more B-FLOPs and that removing them reduces the B-FLOPs and, at the same time, compresses the model significantly. Removing the layers with more B-FLOPs eventually compresses the model; however, removing them redundantly will cause a bigger drop in accuracy. Thus, to maintain accuracy, we only performed minimal pruning.

Comparison of the performance of the pruned models on Dataset B revealed that the YOLOv4-RC3 model has the second-highest recall rate at 95%. In contrast, the YOLOv4-RC4 has the lowest recall rate among the pruned models at 91%. Both these models have the same number of layers, and both attained a precision of 50%. However, the difference in the recall rate between these two models shows that the YOLOv4-RC3 model can generalise better on Dataset B, primarily because YOLOv4-RC3 has smaller B-FLOPs. This result suggests that the prediction of the YOLOv4-RC4 is either impacted by the larger number of B-FLOPs or that the residual blocks removed from the C4 Res-Block body contain essential discriminative features. To further evaluate this possibility, residual blocks from C3 and C4 Res-block bodies were removed for the YOLOv4-RC3_4 model.

Analysis of the YOLOv4-RC3_4 model revealed that this model can generalise better on the unseen Dataset B than the other pruned models, showing the highest mAP of 90.70% (Table 8). This model has a better trade-off between precision and recall rate. In the present study, priority was given to the recall rate to flag as many malaria-infected cells as possible. The YOLOv4-RC3_4 model achieved the highest recall rate of 96% and could detect 708 of the 740 infected cells from the thin blood smear images of Dataset B. Upon comparison with the original and the other modified models, the YOLOv4-RC3_4 model was found to be able to detect the highest number of infected cells on the unseen Dataset B. In contrast to its result on Dataset B, however, the model achieved the lowest mAP on Dataset A relative to the other pruned models. One possible explanation is that since the other pruned models have more layers, they likely started to overfit the training data, which explains their higher performance on Dataset A compared to YOLOv4-RC3_4 and reduced accuracy on the unseen Dataset B.

For YOLOv4-RC3_4, the C3 and C4 residual blocks are pruned simultaneously. As a result, this model has added advantages from the pruning of both the C3 and C4 bodies, such as fewer layers, fewer B-FLOPs and a smaller model size. We interpreted the results as indicating that the original model is designed and suitable for vast datasets and problems. More layers will only increase the complexity of the model and training error, and it possibly overfits faster and reduces its generalisation to new unseen data. The improved accuracy of the YOLOv4-RC3_4 model compared to the two previously pruned models indicates that fewer layers, smaller B-FLOPs and smaller size positively impact the performance of the models on our datase, suggesting that the performance of YOLOv4-RC4 was mainly affected by the high number of B-FLOPs. This was proven upon observation that the model’s accuracy improved upon the removal of residual blocks from both the C3 and C4 Res-block bodies. The improved prediction and generalisation of the model show that the residual blocks removed from the C3 and C4 Res-block bodies were redundant and that their removal did not degrade the model’s performance.

In addition to the simultaneous pruning of the C3 and C4 residual blocks, simultaneous pruning of the C3 and C5 Res-block bodies was also performed. Both the YOLOv4-RC3 and YOLOv4-RC5 models exhibit good prediction accuracy on Dataset A. Therefore, the C3 and C5 residual blocks were pruned simultaneously to evaluate their performance. However, the prediction performance of YOLOv4-RC3_5 decreases on Dataset A. This model can give good predictions on Dataset B, but its degradation in mAP is mainly affected by its lower recall rate. The YOLOv4-RC5 and YOLOv4-RC3_5 models do not differ much in terms of precision and recall rate, indicating that removal of the C3 residual blocks in YOLOv4-RC3_5 does not affect the model’s performance and further suggesting that the residual blocks in the C3 body may not contribute significantly to prediction accuracy and carry fewer discriminative features. Removing these residual blocks did not harm the model's performance. Our analysis shows that the C5 Res-block bodies are more effective at identifying malaria-infected cells due to their stronger discriminative power. This became evident when good prediction accuracy was maintained when these residual blocks are kept.

In previous studies, researchers have experimented with replacing the YOLOv4 model backbone with a lighter network as the feature extractor. For example, Wang et al. [52] demonstrated that the model's accuracy increases upon replacing the backbones with the shallower network of EfficientNet. Our experiments revealed that the models with the ResNet-50 backbone achieved higher precision than the original YOLOv4 models, but with a lower recall rate. This eventually means that the modified model cannot detect many infected cells as true positives compared to the original model. This is a disadvantage in our case, as priority is given to identifying as many infected cells as possible. The pruned models with the CSP-DarkNet-53 backbone can identify more infected cells than those with ResNet-50 backbone, indicating that ResNet-50’s feature extraction is suitable but that it may need to learn more essential features for predicting infected cells on the malaria dataset. On the other hand, CSPDarknet53’s feature extraction is more effective, and although it may be too complex for our dataset, its accuracy can be improved with proper modifications. Our results show that the shallower pruned model with fewer layers and B-FLOPs and smaller size is more generalised. Removing unnecessary residual blocks can reduce the model's size and improve accuracy without replacing the backbone entirely.

### Modifications of YOLO models for malaria diagnosis in other studies

In previous studies closely related to the present study, the YOLO model has been employed for detecting malaria parasites from thick blood smear images [33, 62] and thin blood smear images [30–32]. Abdurahman et al. [62] modified the architecture of the YOLOv4 model by adding a fourth detection layer to the existing network to detect parasites from thick blood smear images. The added network could extract more robust geometric features concatenated with the deep-level features using PANet architecture, with the mAP of the model increasing from 83.64% to 89.73% (Intersection over Union [IoU] = 0.5) following the modification. However, the addition of an extra detection layer caused the size of the model to increase. While this modification effectively improved the detection of malaria parasites in thick blood smear images, the complexity of the model requires improvement. Similarly, Koirala et al. [33] created a customized three-layered YOLO-mp3I and four-layered YOLO-mp4I to detect parasites from thick blood smear images with a mAP of 93.99% (IoU = 0.5) and 94.07% (IoU = 0.5). The modified lightweight models outperformed the original YOLOv4 model with fewer B-FLOPs and smaller model size.

For the application of object detectors on thin blood smear images, Yang et al. [30] proposed a cascaded YOLOv2 model to detect malaria-infected cells. The YOLOv2 model was used to detect infected cells and the AlexNet classifier was used to reduce the number of false positives. Although the accuracy of the cascaded model improved to 79.22% relative to the original model, there is still room for improvement in terms of accuracy. The relatively lower accuracy for the detection of only *P. vivax*-infected cells questions the robustness of the model to detect cells infected by other malaria parasite species and from an unseen dataset. Therefore, investigations on the use of additional object detectors and the YOLO model on thin blood smear images remain worthwhile.

To the best of our knowledge, the present study is the first to train, modify and optimise the YOLOv4 model to detect malaria-infected cells from whole thin blood smear images. In our study, the original YOLOv4 and pruned YOLOv4-RC3_4 models outperform the cascaded YOLOv2 model [30] in detecting malaria-infected cells from whole thin blood smear images. It is worth highlighting that our models deals with images infected by all malaria parasites to train, test and cross-validate the models. Despite this, the model is able to achieve a satisfying prediction accuracy.

The performance of the models varies when performing cross-dataset testing, which ultimately indicates that the model’s performance is prone to changes upon testing a new unseen dataset. Testing the models on an independent dataset provides a better overview of the model’s generalisation and robustness. We interpreted these results as indicating that a model may perform well on images that resemble the training images, but that the accuracy might vary, indicating possible overfitting and training error. For the present study, information on the different stains used is a main factor that affects the models' predictions in an unseen dataset. However, this problem may be uncontrollable as the staining may vary in different settings. One possible solution is to increase the variety in the training images instead of limiting them to images with the same stain information. This approach can help the model learn the features of the infected cells despite differences in staining information.

The main conclusion that can be drawn from this study is that using deep-learning object detectors for the detection of malaria-infected cells is relatively advantageous. In previous studies, the CNN models were mainly used to classify the cells as infected or non-infected. These models did achieve impressive results in terms of classifying the cells. For example, Sriporn et al. [18] reported that the best-performing CNN model achieved an accuracy of 99.98% in classifying infected and non-infected cells. However, for a real-time application, it is not feasible to feed single-cell images into a network to identify whether they are infected or non-infected. Rather, in a real-time application, it is only practical to use the whole thin blood smear image to detect the infected cells, and the object detectors make this applicable.

To this point, we have proven that deep learning object detectors indeed can contribute to a more efficient automatic malaria diagnosis. However, a number of constraints are associated with object detection, one of which pertains to the computational expense involved in training the entire thin blood smear image. Moreover, the utilisation of whole-thin blood smear images amplifies the probability of erroneous positive predictions within the image owing to the inaccurate depiction of stains on the non-infected cells. A situation akin to this was documented by Yang et al. [30], and it is possible that utilising an entire thin blood smear image may result in several false positive results. That being said, there are ways to handle this situation.

In the future, infected cells and other stained materials within the thin blood smear image can be predicted and classified by the object detectors into two distinct groups. This may gradually lower the number of false-positive predictions by the models and increase their dependability in real-world scenarios. However, the advantages of using object detectors outweigh their drawbacks. It is possible to avoid depending on individual cell photos by using object detectors. The single-cell photos can be simultaneously cropped and prepared with the expected bounding boxes. In the suggested method, single-cell images that have been cropped can be analysed and classified using a CNN model. The novelties of this research have several potential impacts in a clinical setting, as reported below.

#### Lightweight YOLOv4 for automated malaria detection

Modifying the YOLOv4 model with direct pruning of residual blocks improves the detection of malaria-infected cells. This step may be helpful in a clinical setting where the accurate and efficient identification of malaria cells is essential for making decisions regarding diagnosis and treatment. Layer pruning improves malaria diagnosis by reducing model size, complexity and inference time, resulting in faster and more efficient automatic detection. Developing lighter variants of deep learning models, optimising the architecture of models for specific diagnostic tasks and taking into account the computational needs of edge devices and low-resource environments are required to address this research problem. By refining complex algorithms, researchers can ensure that the benefits of advanced technology are accessible to a larger population and can be utilised effectively in the fight against malaria. Pruned YOLOv4 models have improved generalisation and robustness, making them extraordinarily adept in detecting cells infected by various malaria parasite species. This improvement in model performance considerably enhances the capacity of diagnostic systems to deliver consistent, dependable and accurate results across a broad range of clinical scenarios. By strategically removing layers, the model is simplified, allowing it to operate seamlessly on clinical devices with limited resources. By optimising model complexity, we can enable healthcare providers to provide more effective care, ensuring that patients receive timely interventions regardless of the complexities posed by combined infections or malaria caused by diverse parasite species.

#### Recognising the malaria species diversity

The primary focus of the present study was to detect malaria-infected cells rather than species identification. However, to ensure the robustness and generalisation of the developed model, we intentionally introduced multiple species of malaria parasites during the training and testing phases. By including images of cells infected by a variety of malaria parasites, including* P. knowlesi*, an emerging zoonotic malaria parasite, our research takes a unique and crucial perspective in directly detecting infected cells from whole thin blood smear images, regardless of the specific malaria species present. In addition, the focus of our study differs from that typically found in previous studies in that we included a wide range of malaria species, rather than limiting the study to a limited number of well-known human malaria species. This approach is significant as it allows our proposed model to identify infected cells despite the diversity of malaria parasites present in the images. This study design also ensures the proposed model is equipped to effectively identify and localise infected cells in diverse clinical scenarios. Our comprehensive approach not only enhances the model's performance but also strengthens its applicability in automatic diagnostic setting where mixed infections are prevalent.

#### Automated localisation of infected cells

In the present study, we explored the notion of automatically cropping the detections to make single-cell images, in addition to employing object detectors to identify the infected cells. A major barrier to training CNN models is the scarcity and availability of single-cell images, and automated segmentation single-cell images using conventional segmentation techniques is a difficult task. Thus, in this work, the cell cropping was accomplished using the bounding box coordinates (left_x, top_y, width and height) that are created following detections. This step facilitates cropping and the production of single-cell images, and it creates the opportunity to study and train CNN models for other classification problems using bounded cell images rather than segmented ones.

### Future work

**Fig. 9:**
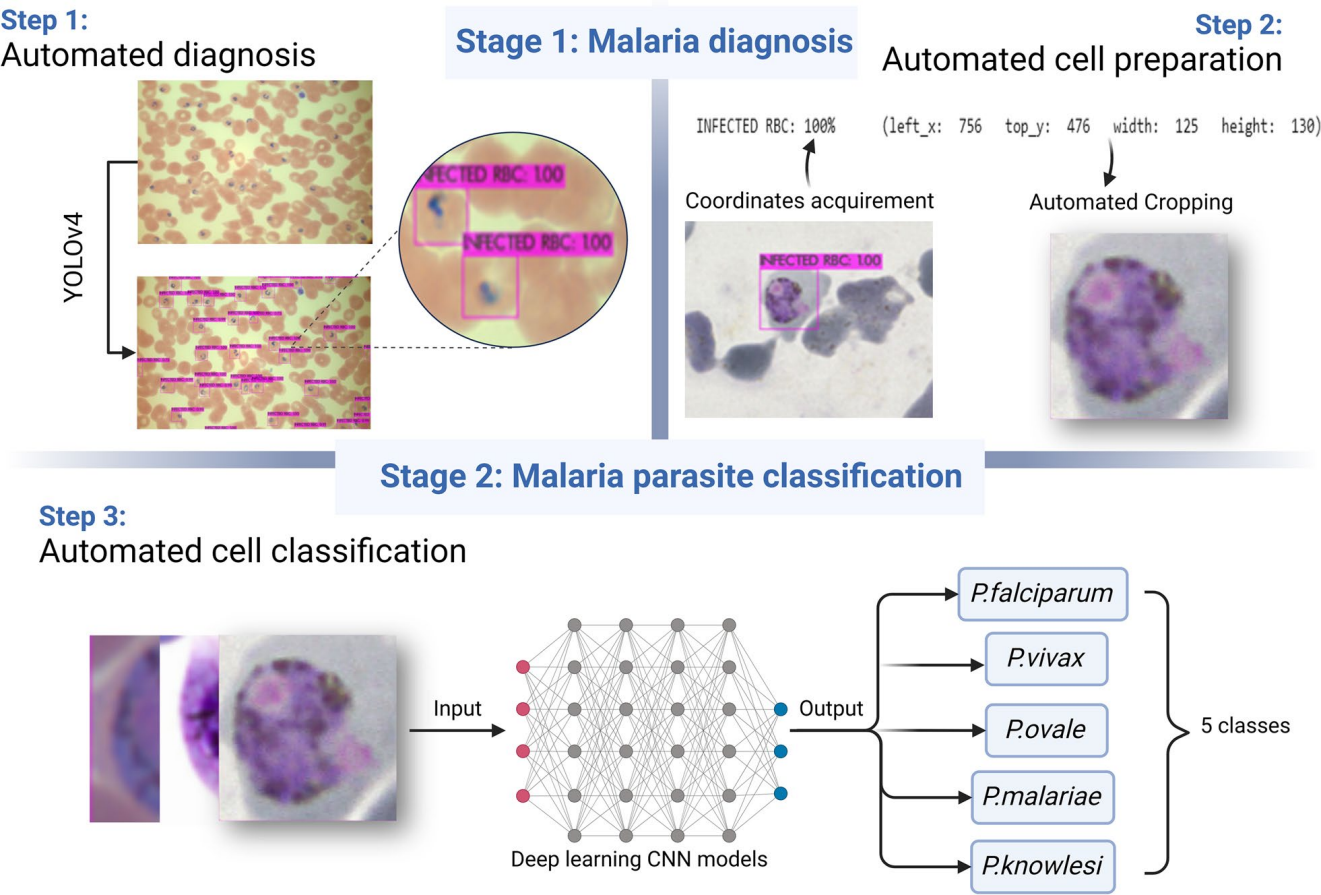
Overall framework of proposed automated malaria diagnosis and species identification. CNN, Convolutional neural network; RBC, red blood cell; YOLO, You Only Look Once (model)

Future steps in this research entail training different CNN models with the automatically cropped, bounded single-cell images in order to categorise the images based on a specific parasite (*P. falciparum*,* P. vivax*,* P. malariae*,* P. ovale*,* P. knowlesi*) that causes the infection (Fig. 9). Employing CNN models for the classification of single-cell images according to a specific malaria parasite species is more ideal than using them for malaria diagnosis. The overall aim is, therefore, to use object detectors for diagnosis and single-cell preparation, whereas the CNN models perform classification of the single-cells according to their parasitic infection. Different parasite species may respond differently to antimalarial medications. Therefore, determining the precise parasite infection is essential because it allows drugs to be administered that are suited to treatment of the particular species identified, resulting in the best possible care for the patient. In addition, in the actual world, patients can be infected by multiple parasite species or strains simultaneously. Thus, detecting mixed infections is vital for ensuring appropriate treatment strategies. A major development in the thorough diagnosis and treatment of malaria can be seen in the increasing breadth of species coverage and classification. Deep learning applications may become more end-to-end and practical in real-time healthcare settings in the near future by utilising object detectors for diagnostics and CNN models for parasite species identification.

## Conclusions

The results of this study represent a pivotal advancement in the field of automated malaria diagnosis using deep learning models, specifically the YOLOv4 architecture. Our research has demonstrated the potential to simplify complex computational algorithms through strategic layer pruning, making the model more lightweight and efficient. This novel approach improves the generalisation and robustness of YOLOv4 to detect cells infected by all malaria parasites species and from an unseen dataset. The original YOLOv4 model demonstrated satisfactory results in predicting cells. The layer pruning approach and backbone replacement method were compared to develop a shallower model. We found that decreasing the number of layers improves model learning but that over-pruning should be avoided to prevent difficulty in learning. We also found that residual block pruning is a technique that can enhance model accuracy while reducing complexity. This approach is viable and keeps the model's performance intact. In addition, the simplified model's accessibility to clinical devices with limited resources expedites malaria detection and facilitates prompt treatment, particularly in regions with limited medical facilities. By addressing these critical issues and proposing novel solutions, our research paves the way for the deployment of deep learning models in the fight against malaria in a manner that is both practical and effective. As we continue to aspire for more effective and accessible malaria diagnosis, our work exemplifies the potential for computational innovations to have a substantial impact on global healthcare initiatives.

## Data Availability

The source of data acquired is cited in the text in the Methods section. Dataset A supporting the conclusions of this article is available at https://github.com/andrealoddo/MP-IDB-The-Malaria-Parasite-Image-Database-for-Image-Processing-and-Analysis. Dataset B can be obtained from the Malaria Research Centre, UNIMAS.

## Abbreviations

B-FLOPS: Billion floating-point operations
CBM: Convolutional, Batch normalisation and Mish
CNN: Convolutional neural network
mAP: Mean average precision
RBC: Red blood cell
